# Metastatic breast cancers have reduced immune cell recruitment but harbor increased macrophages relative to their matched primary tumors

**DOI:** 10.1186/s40425-019-0755-1

**Published:** 2019-10-18

**Authors:** Li Zhu, Jessica L. Narloch, Sayali Onkar, Marion Joy, Gloria Broadwater, Catherine Luedke, Allison Hall, Rim Kim, Katherine Pogue-Geile, Sarah Sammons, Naema Nayyar, Ugonma Chukwueke, Priscilla K. Brastianos, Carey K. Anders, Adam C. Soloff, Dario A. A. Vignali, George C. Tseng, Leisha A. Emens, Peter C. Lucas, Kimberly L. Blackwell, Steffi Oesterreich, Adrian V. Lee

**Affiliations:** 1 Department of Biostatistics, University of Pittsburgh, Pittsburgh, PA USA; 2 Clinical Research Training Program, Duke University Medical Center (DUMC), Durham, NC USA; 3 Breast Cancer Program, Duke Cancer Institute, DUMC, Durham, NC USA; 4 Department of Immunology, University of Pittsburgh, Pittsburgh, PA USA; 5 Tumor Microenvironment Center, University of Pittsburgh Medical Center (UPMC) Hillman Cancer Center, Pittsburgh, PA USA; 6 National Surgical Adjuvant Breast and Bowel Project (NSABP), Pittsburgh, PA USA; 7 Duke Clinical Research Institute, Durham, NC USA; 8 Department of Pathology, DUMC, Durham, NC USA; 9 Division of Hematology/Oncology, Department of Medicine, DUMC, Durham, NC USA; 10 Division of Hematology & Oncology, Department of Medicine, Massachusetts General Hospital, Harvard Medical School, Boston, MA USA; 11 Division of Neuro-Oncology, Department of Neurology, Massachusetts General Hospital, Harvard Medical School, Boston, MA USA; 12 Division of Medical Oncology, Department of Medicine, University of North Carolina at Chapel Hill, Chapel Hill, NC USA; 13 Department of Cardiothoracic Surgery, University of Pittsburgh School of Medicine, Pittsburgh, PA USA; 14 Department of Medicine, University of Pittsburgh, Pittsburgh, PA USA; 15 Womens Cancer Research Center, UPMC Hillman Cancer Center, Magee Womens Research Institute, Pittsburgh, USA; 16 Department of Pathology, University of Pittsburgh School of Medicine, Pittsburgh, PA USA; 17 Department of Radiation Oncology, DUMC, Durham, NC USA; 18 Department of Pharmacology & Chemical Biology, University of Pittsburgh, Pittsburgh, USA

**Keywords:** Metastatic breast cancer, Breast cancer, Macrophages, M2 macrophages

## Abstract

The interplay between the immune system and tumor progression is well recognized. However, current human breast cancer immunophenotyping studies are mostly focused on primary tumors with metastatic breast cancer lesions remaining largely understudied. To address this gap, we examined exome-capture RNA sequencing data from 50 primary breast tumors (PBTs) and their patient-matched metastatic tumors (METs) in brain, ovary, bone and gastrointestinal tract. We used gene expression signatures as surrogates for tumor infiltrating lymphocytes (TILs) and compared TIL patterns in PBTs and METs. Enrichment analysis and deconvolution methods both revealed that METs had a significantly lower abundance of total immune cells, including CD8+ T cells, regulatory T cells and dendritic cells. An exception was M2-like macrophages, which were significantly higher in METs across the organ sites examined. Multiplex immunohistochemistry results were consistent with data from the in-silico analysis and showed increased macrophages in METs. We confirmed the finding of a significant reduction in immune cells in brain METs (BRMs) by pathologic assessment of TILs in a set of 49 patient-matched pairs of PBT/BRMs. These findings indicate that METs have an overall lower infiltration of immune cells relative to their matched PBTs, possibly due to immune escape. RNAseq analysis suggests that the relative levels of M2-like macrophages are increased in METs, and their potential role in promoting breast cancer metastasis warrants further study.

## Introduction

Breast cancer is a highly heterogenous disease affecting 1 in 8 women in the US, and the most commonly diagnosed cancer in women worldwide. Despite recent improvements in overall survival rates, it is still the second leading cause of mortality due to cancer in women [1]. In the last two decades, significant progress has been made in the detection and treatment of primary breast tumors as a result of enhanced understanding of disease biology and the tumor microenvironment (TME). The breast TME represents a complex interaction between tumor cells, endothelial cells, fibroblasts, and a variety of pro- and anti-tumor immune cells capable of tipping tumor biology toward tumor growth and progression or immune rejection. During tumor growth, cancer cells can be detected and eliminated by the immune system, but some cancer cells may exploit several mechanisms to evade destruction by the immune system, enabling them to escape immune surveillance and progress through the metastatic cascade. For breast cancer, the most common sites of distant organ metastases include bones, lungs, liver and brain with ovaries and gastrointestinal tract (GI) being affected less frequently [2].

The interplay between the immune system and tumor development is now well recognized in a variety of tumor types, including the triple negative (TNBC) and HER2+ subtypes of breast cancer [3, 4]. However, existing immunophenotyping studies focus mainly on primary tumors, with the role of immune cells in metastatic progression remaining largely understudied. While numerous studies have now documented cellular and genomic evolution of breast cancers during metastasis [5, 6], very little is known about the co-evolution of immune cells and the TME. This study focused on addressing this gap in our understanding by performing immunophenotyping on two datasets: a) Pan-MET, transcriptomic profiles of 50 pairs of patient-matched primary (PBTs) and metastatic breast tumors (METs) in brain (BRM), ovary (OVM), bone (BOM) and gastrointestinal tract (GIM); and b) BRM-sTIL, a multi-institutional cohort of 49 patient-matched pairs of PBTs and BRMs with stromal tumor infiltrating lymphocytes (sTILs) percentages quantified by pathologic evaluation of hematoxylin & eosin (H&E) staining. Using gene expression signatures as surrogates for TILs, we discovered quantitative differences in immune cell profiles between PBTs and METs in the first dataset (Pan-MET). Those differences were confirmed using multiplexed immunofluoresence (mIF) in three pairs of PBT/OVMs and PBT/BRMs each. Consistent results were observed by comparing the sTILs percentages in additional PBT/BRM pairs in a second dataset (BRM-sTIL). Higher immune cell recruitment to the TME also showed weak association with better survivals in both datasets. Our study demonstrates the potential of using bioinformatics tools to investigate the evolution of the immune TME in breast cancer metastasis, and identifies M2-like macrophages as a potential therapeutic target for metastatic breast cancer.

## Materials and methods

Details of methods are available in Additional file 1.

### Data

#### Pan-MET dataset

Exome-capture RNA sequencing (ecRNA-seq) of patient-matched PBTs and METs were collected from brain, bone, ovary and GI, as previously reported in [7–9]. Clinical and pathological information of all samples are available in Additional file 2: Table S1. Formalin fixed paraffin embedded (FFPE) tissue sections of three pairs of PBT/BRMs and PBT/OVMs each were retrieved from the Pitt Biospecimen Core for multiplex staining.

#### BRM-sTIL dataset

Sample tissues of 49 pairs of patient-matched PBTs and BRMs were collected from four participating academic institutions (Duke University Medical Center, University of North Carolina Medical Center, University of Pittsburgh, Massachusetts General Hospital) for H&E staining. Clinical and pathological information is available in Additional file 2: Table S2. 15 pairs of PBT/BRMs overlap between the Pan-MET and BRM-sTIL (Additional file 2: Table S3).

### Immune level quantification

We inferred the immune abundance from RNAseq data using single-sample gene set enrichment analysis (ssGSEA, i.e. immune score in ESTIMATE) [10], gene set variation analysis (GSVA) [11] and deconvolution methods --- CIBERSORT [12] and TIMER [13]. In addition to the samples in Pan-MET dataset, we also evaluated immune level in normal tissue samples achived from Genotype-Tissue Expression (GTEx) Project. H&E stained sections in BRM-sTIL dataset were manually counted for percent sTILs using standard criteria developed by the international TILs working group [14]. Each slide was independently reviewed by two study personnel (JLN and CL) to minimize inter-observer variability. When the sTILs differed by 10% or more, the study pathologist (AH) made the final determination.

## Results

### METs have lower total immune abundance than patient-matched PBTs

**Fig. 1:**
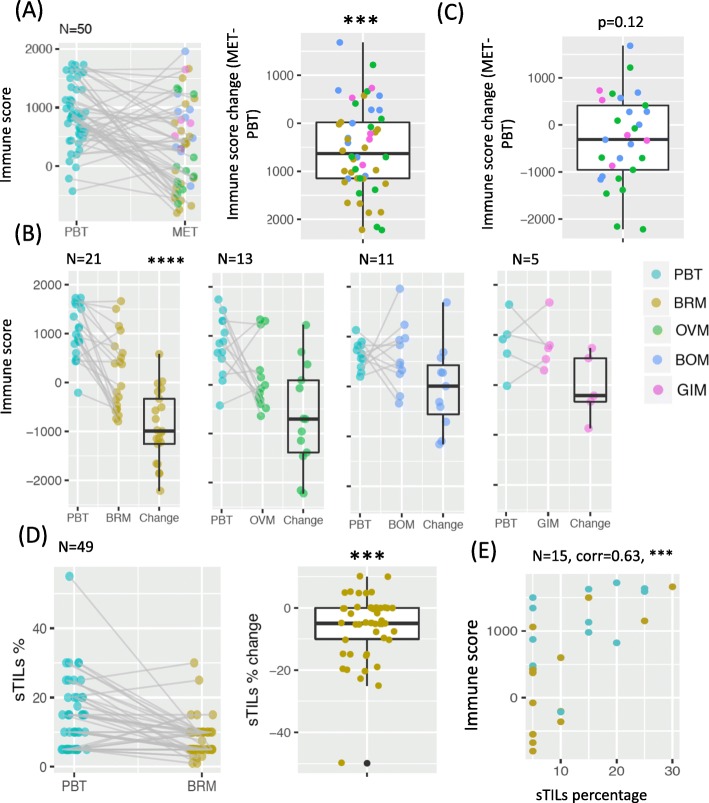
Lower immune abundance in metastatic breast tumors (METs) compared to primary breast tumors (PBTs) (**a**) Total immune score in PBT/MET pairs in Pan-MET dataset, together with the paired changes (MET-PBT). **b** Paired changes of total immune score removing BRMs in (**a**). **c** Total immune score grouped by MET sites. **d** Stromal tumor infiltrating lymphocytes (sTILs) percentages of 49 pairs of PBT/BRMs in BRM-sTIL dataset. **e** Spearman’s correlation between sTILs percentages and total immune score for 15 pairs of PBT/BRMs overlapped by Pan-MET and BRM-sTIL. ****p < 0.0001, ***p < 0.001, **p < 0.01, *p < 0.05 from two-sided Wilcoxon signed rank test in (**a**-**d**) and correlation test in (**e**)

We estimated total immune abundance using RNAseq from 50 pairs of patient-matched PBTs and METs. For multiple METs that were matched to the same PBT, we first took the average. In general, METs showed a significantly lower total immune score compared to patient-matched PBTs (Fig. 1a; *p* < 0.001). The decrease in immune score was observed in METs collected from various sites, but was especially apparent in BRMs (*p* < 0.0001, Fig. 1b). Removing BRMs and combing all other METs, we noted a non-significant trend to decreased immune score in METs (*p* = 0.12, Fig. 1c). However, it should be noted that the small number of samples makes conclusions in the non-brain METs challenging. Validating the finding of decreased immune cells in brain METs, pathologic assessment of sTILs in an additional cohort of 49 patient-matched PBTs and METs revealed that BRMs also showed a significant decrease in the percentage of sTILs compared to patient-matched PBTs (*p* < 0.001, Fig. 1d). When grouping PBT/MET pairs by hormone receptor (HR) status and HER2 status, both datasets revealed a trend of decreased immune abundance in all subtypes, with TNBC subtype having the most significant decrease (*p* < 0.01, Additional file 2: Figure S1). Similar results were observed when we treated those METs matched to the same PBT as METs in different pairs (Additional file 2: Figure S2). While the total immune score only estimates the overall immune abundance in the bulk sample from RNAseq, and the sTILs percentage was carefully counted as the immune cell percentage in the stroma, the two measurements of immune abundance were significantly correlated (*p* < 0.001) for the 15 pairs of PBT/BRMs within both data sets (Fig. 1e). A lesser degree of agreement was only observed in tumors with extreme low sTILs (5%), possibly due to unstable estimates by both methods when the immune component is limited.

In addition, we also observed that METs had significantly lower expression of immune checkpoint molecules that downregulate immune response — including CD274 (PD-L1), PDCD1 (PD-1), CTLA4, but not VSIR (Additional file 2: Figure S3) — possibly due to fewer total immune cells. We also tested for differentially expressed (DE) genes between matched PBT/BRMs (ER+ and ER- separately), PBT/OVMs (ER+ only) and PBT/BOMs (ER + only) to eliminate possible confounding effect from ER status. Pathway enrichment analysis of DE genes (adjusted *p* < 0.05) from matched PBT/BRM, both ER+ and ER-, identified immune related pathways, such as KEGG_primary_immunodeficiency, as one of the top significantly enriched pathways (Additional file 3: Table S4, Additional file 4: Table S5). Several immune related pathways were also significantly enriched in PBT/OVM and PBT/BOM comparisons, but they were not among the top 50 significant list (Additional file 5: Table S6, Additional file 6: Table S7).

Taken together, both transcriptomic data and pathological assessment showed that METs have lower immune abundance than patient-matched PBTs.

### METs have higher percentage of M2-like macrophages relative to the total immune abundance

We inferred the abundance of each immune cell population by two types of methods — enrichment analysis and deconvolution method. To validate those approaches, we first compared the GSVA scores of four common immune cell populations defined by both Davoli et al. [15] and Tamborero et al. [16]. The correlations ranged from 0.4 to 0.85 (Additional file 2: Figure S4), indicating overall high consistency. For further validation, we applied four methods; namely GSVA using the immune signatures from Davoli and Tomborero, and two methods of deconvolution (CIBERSORT and TIMER) to a publicly available single cell RNA-seq dataset [17], in which immune cell percentages were available using cell markers. Based on the correlations, the estimated levels of B cell, T cell, and macrophages by immune signatures from Davoli and Tamborero, and deconvolution method TIMER, were in general most highly correlated with actual abundance of corresponding cell types, although some signatures were not quite specific, such as CD4+ mature T cell and CD8+ effector T cell in Davoli signatures. CIBERSORT estimates showed lower correlations as expected, because the actual percentages were calculated based on three cell types, while CIBERSORT considered 22 cell types (Additional file 2: Figure S5).

**Fig. 2:**
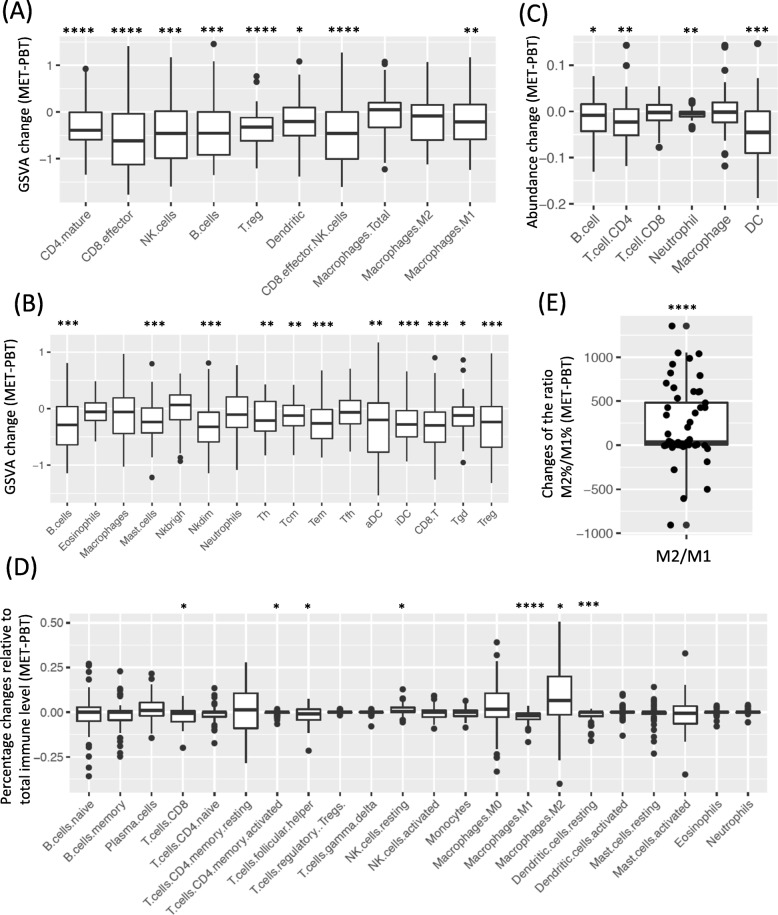
Paired comparison of the abundance of immune cell population in PBT/MET pairs in Pan-MET. **a**-**b** GSVA score changes (MET-PBT) of (**a**) Davoli signature and (**b**) Tamborero signature. **c** Abundance changes estimated by deconvolution method TIMER. **d** Changes of percentages relative to total immune level estimated by deconvolution method CIBERSORT. **e** Changes of the ratio of relative percentages of M2 and M1. ****FDR < 0.0001, ***FDR < 0.001, **FDR < 0.01, *FDR < 0.05 by Benjamini-Hochberg correction. Two-sided Wilcoxon signed rank test

Comparing patient-matched PBTs and METs, the GSVA score and abundance estimate from deconvolution methods for most immune cell populations were significantly lower in METs (Fig. 2a-c). Adjusting for total immune abundance, most immune cell populations were still lower, but M2-like macrophages were significantly higher in METs (Fig. 2d). Since CIBERSORT provides an empirical *p* value testing the null hypothesis that a particular sample does not contain any of the 22 cell types, we removed 16 pairs with at least one sample with *p* > 0.05, M2-like macrophages were still higher in METs, but there was only a trend to significance (Additional file 2: Figure S6). Significant increment was also observed in the ratio of the relative percentages of M2 and M1, indicating dominant level of M2 over M1 (Fig. 2e). When separating PBT/MET pairs to different MET sites or HR/HER2 subtypes, the results were generally consistent (Additional file 2: Figure S7-S8). Due to the lack of adjacent normal tissues, it is impossible to fully eliminate the effect contributed by the different cellular composition of the normal tissues. However, when comparing the percentage of M2-like macrophages in normal tissues with RNAseq data downloaded from GTEx, we observed that M2 macrophages was lower in normal brain and small intestine and similar in ovary (normal bone tissue is not available in GTEx) compared to normal breast, suggesting that the increased M2 macrophage in METs was not due to the presence of normal tissues (Additional file 2: Figure S9).

### Multiplexed immunofluoresence confirms the in-silico results

**Fig. 3:**
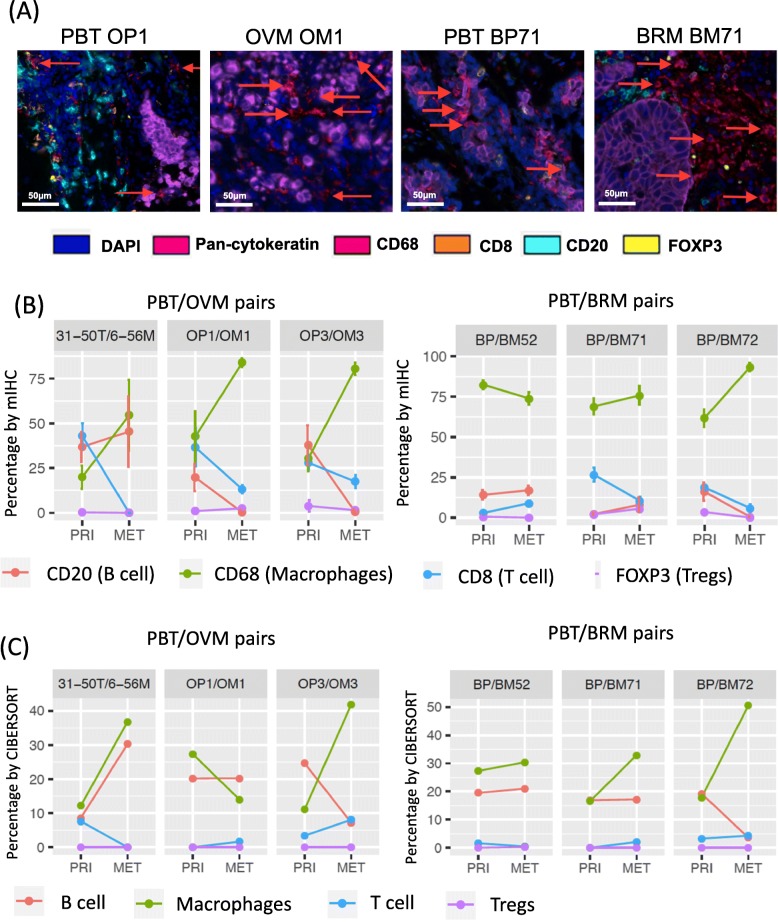
Multispectral immunohistochemical (mIHC) staining of selective pairs in Pan-MET. **a** mIHC staining images of one pair of PBT/OVMs and PBT/BRMs. **b** Percentage (by cell) of each immune cell population denoted by markers using mIHC staining. **c** Relative percentages of corresponding immune cell populations estimated by CIBEROSRT

To further validate in silico results, we selected three pairs of PBT/BRMs and three pairs of PBT/OVMs, which were shown to have higher M2-like macrophages relative to the total immune abundance, for multispectral immunofluorescence (Fig. 3a). Three pairs of PBT/OVMs and two pairs of PBT/BRMs showed increased macrophages in METs, and the majority of METs had lower B cells and T cells (Fig. 3b), consistent with percentage estimated from CIBERSORT (Fig. 3c and Additional file 2: Figure S10).

### Hormone receptor (HR) positive tumors are associated with lower total immune abundance

**Fig. 4:**
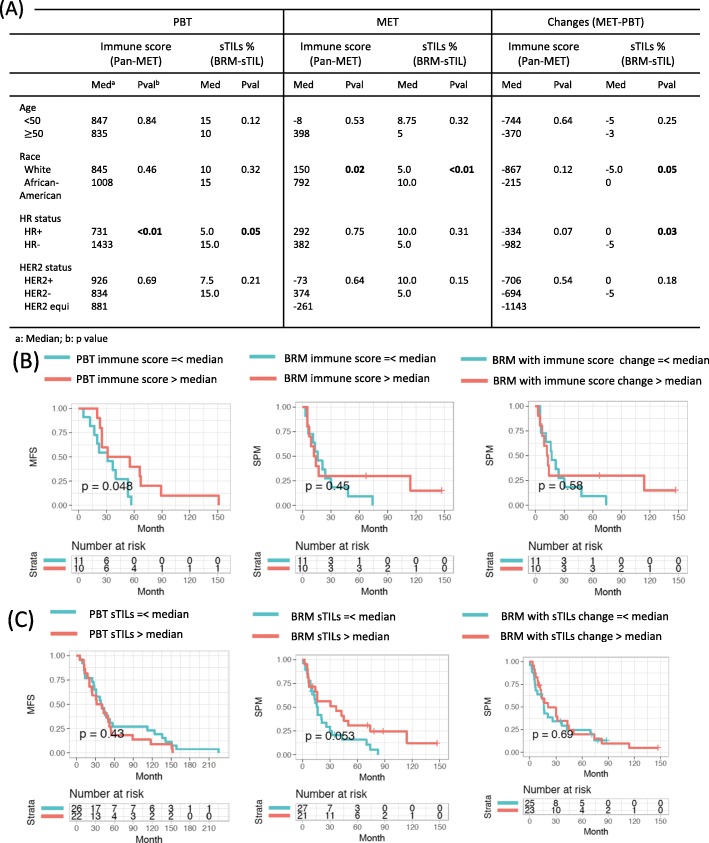
Association of immune abundance with clinical variables and survivals. **a** Association between immune score and sTILs with clinical variables. **b** Association between survivals and immune score of PBT/BRM pairs in (**b**) Pan-MET dataset and (**c**) BRM-sTIL dataset. ****p < 0.0001, ***p < 0.001, **p < 0.01, *p < 0.05 from Wilcoxon signed rank and Kruskal-Wallis test in (**a**) and log-rank test in (**b**)-(**c**)

To examine the contribution of each clinical variable, we tested the association between immune level (at PBT, MET and their changes) and all clinical variables available (Additional file 7: Table S8, Additional file 8: Table S9). Both the RNAseq and the sTIL dataset revealed that HR+ PBTs have significantly lower immune scores than HR- PBTs (Fig. 4a). Further, HR+ METs tended to have a smaller decrease in immune abundance compared to PBTs, although this was only significant in the BRM-sTIL dataset. However, stratifying tumors by HR and HER2 status revealed that METs in all categories had lower immune level than paired PBTs (Additional file 2: Figure S1), indicating that decreased immune is not entirely due to HR status. On the other hand, therapies were also strongly associated with the immune level, but they were highly related to tumor subtypes − 94% of ER+ cases received endocrine therapy; 64% HER2+ cases and 6% HER2- patients received HER2 treatment; 87% of all cases received chemotherapy. Due to the heterogeneity of the treatments, and the association with subtype, it is not possible to correct for this confounding variable.

### Higher immune abundance is weakly associated with longer time to development of BRMs and longer survival post BRMs

We hypothesized that immune level of PBT may be associated with metastasis-free-survival (MFS), while immune level of MET and its change from PBT to MET are potentially associated with survival-post-metastasis (SPM). Combining all PBT/MET pairs into one cohort, immune score was not significantly associated with MFS or SPM (Additional file 2: Figure S11), likely due to the confounding effect of different MET sites on outcome. Considering PBT/BRM pairs had the largest sample size, we tested the potential association between immune score and survival specifically in PBT/BRMs. In the pan-MET dataset, there was a trend in association between higher immune levels in PBTs and longer time to development of BRMs (i.e. MFS) (Fig. 4b). However, such a trend was not observed between SPM with immune levels in BRM or immune level change between PBT and BRM (Fig. 4b). In the BRM-sTIL dataset, higher sTILs percentage in PBT was not associated with MFS. Instead, there was a trend toward an association between a higher sTILs percentage in MET and longer SPM (Fig. 4c). We did not observe significant associations between the relative level of M2-like macrophage and survivals (Additional file 2: Figure S12).

## Discussion

It is now well appreciated that immune cells are a critical component of the TME. Studies of the breast TME have largely focused on tumor mutational and transcriptional landscapes in primary breast cancers, and with more recent attention to metastatic tumors. Our study is novel in two main regards: (1) we examined two cohorts of matched PBTs and METs, one of which includes METs in different sites, allowing us to discern site-specific immune changes from primary to metastatic disease and (2) we evaluated immune abundance by both gene expression analysis and H&E staining, and observed overall high consistency. Our data demonstrate the potential of using bioinformatics tools to investigate the immune contexture of both primary and matched metastatic tumors when tumor lesions may not be available for staining.

Our paired patient-matched comparison revealed a decrease in immune cells from primary to metastatic breast cancer, which is consistent with limited existing studies [18–20]. In-silico analysis of the Pan-MET dataset, validated by mIF staining, highlights the potential enrichment of M2-like macrophages as the tumor cells metastasize to various sites, especially brain and ovary. This is consistent with the growing body of literature that has shown macrophages to be one of the key players in establishment of distant METs [21–23]. Our survival analysis suggests enhanced MFS and SPM in patients with higher immune cell recruitment to primary and metastatic tumors, although the significance of these findings were not consistent between the Pan-MET and BRM-sTIL, possibly due to small sample size and/or sample heterogeneity.

This work has multiple important strengths. First, it utilizes established genomic data sets for elucidating the immunobiology of matched PBTs and METs. Second, it is one of the larger studies of a cohort of patient-matched PBTs and METs. Third, it effectively integrates state-of-the-art genomic analyses with multiplexed immunohistochemistry conducted in a subset of tumors to confirm results. Our study also has several limitations. First, due to the scarcity of patient-matched pairs of primary and metastatic breast cancer, our sample set remains somewhat small relative to studies of primary breast tumors alone. Second, RNAseq analysis was performed on bulk tumor samples, and thus gene expression cannot be attributed to specific cells. Although we attempted to reduce such bias by normalizing the immune score against the non-tumor cell percentage (with consistent conclusions), single cell RNA-sequencing may be needed to completely resolve uncertainties related to cellular heterogeneity. Third, in our mIF studies, the percentage of all immune cells within the tumor was often below 10%. Given these limited numbers of immune cells, our results should be interpreted with caution. Despite these limitations, our study clearly highlights an opportunity to utilize existing data to shed light on the co-evolution and involvement of immune cells in the progression of a primary tumor and its metastatic cascade within an individual patient. It also nominates M2-like macrophages as a potential target for therapeutic immune manipulation of the metastatic cascade.

## Supplementary information

**Additional file 1.** Details of methods.

**Additional file 2: Table S1.** Clinical information of samples in Pan-MET dataset. **Table S2.** Clinical information of samples in BRM-sTIL dataset. **Table S3.** 15 pairs of PBT/BRMs overlap between the Pan-MET and BRM-sTIL. **Table S10.** Detailed list of antibodies and dilutions used for multispectral immunofluorescence staining of slides as shown in Fig. 3. **Figure S1.** Comparison of immune abundance in metastatic breast tumors (METs) and primary breast tumors (PBTs) grouped by HR/HER2 subtypes. (A) Total Immune score in Pan-MET dataset, together with the paired changes (MET-PBT). (B) sTILs percentages of PBT/BRM pairs in BRM-sTIL dataset, together with the paired changes (MET-PBT). ****p < 0.0001, ***p < 0.001, **p < 0.01, *p < 0.05 from two-sided Wilcoxon signed rank test. **Figure S2.** Comparison of immune abundance in METs and PBTs, treating multiple METs matched to the same PBT as MET in different pairs. (A) Total immune score in PBT/MET pairs in Pan-MET dataset. (B) Total immune score grouped by MET sites. (C) Total immune score in Pan-MET dataset grouped by HR/HER2 subtypes. ****p < 0.0001, ***p < 0.001, **p < 0.01, *p < 0.05 from two-sided Wilcoxon signed rank test in (A-C) and correlation test in (D). **Figure S3.** Expression (log2(TPM + 1)) of CD274 (PD-L1), PDCD1 (PD-1), and CTAL4 in PBT and MET. Two-sided Wilcoxon signed rank test was used to compare PBT and MET. Spearman’s correlation with immune score change was calculated and tested using correlation test. ****p < 0.0001, ***p < 0.001, **p < 0.01, *p < 0.05. **Figure S4.** Correlation between GSVA scores of Davoli and Tamborero signatures for PBT/MET pairs in Pan-MET dataset. **Figure S5.** Correlation between immune abundance estimated from RNA-seq data and cell count/proportion (relative to total immune cell count) in single cell RNA-seq dataset. (A-B) GSVA score of (A) Davoli and (B) Tamborero signatures. (C) Percentage relative to total immune level estimated by CIBERSORT. (D) Immune abundance estimated by TIMER. White in the heatmap indicates CIBERSORT estimates are all zero, and spearman’s correlation is not applicable. **Figure S6.** Changes of percentages relative to total immune level estimated by deconvolution method CIBERSORT. METs matched to the sample PBT were treated as different pairs. 16 pairs with at least one sample with *p* > 0.05 were removed from the comparison. **Figure S7.** Comparison of the abundance of immune cell population in PBT/MET pairs grouped by MET sites in Pan-MET dataset. (A-B) GSVA score change (MET-PBT) of (A) Davoli and (B) Tamborero signatures. (C) Abundance change estimated by deconvolution method TIMER. (D) Change of percentage relative to total immune estimated by deconvolution method CIBERSORT. ****FDR < 0.0001, ***FDR < 0.001, **FDR < 0.01, *FDR < 0.05. Two-sided Wilcoxon signed rank test. **Figure S8.** Comparison of the abundance of immune cell population in PBT/BRM pairs grouped by HR/HER2 in Pan-MET dataset. (A-B) GSVA score change (BRM-PBT) of (A) Davoli and (B) Tamborero signatures. (C) Abundance change estimated by deconvolution method TIMER. (D) Change of percentage relative to total immune estimated by deconvolution method CIBERSORT. ****FDR < 0.0001, ***FDR < 0.001, **FDR < 0.01, *FDR < 0.05. Two-sided Wilcoxon signed rank test. **Figure S9.** Comparison of M2-like macrophages percentage in normal brain, breast, ovary and small intestine tissues. RNA-seq data (TPM) were downloaded from GTEx. *N* = 100 samples were randomly selected from each tissue. **Figure S10.** Correlation between mIHC staining results and CIBERSORT estimates. (A) PBT/OVM pairs and (B) PBT/BRM pairs in Pan-MET. Spearman’s correlation. **Figure S11.** Test association between survivals and total immune score of all pairs of PBT/METs in Pan-MET dataset. (A) Kaplan-Meier (KM) curves of MFS for PBTs with total immune score below or above median. (B) KM curves of SPM for METs with total immune score below or above median. (C) KM curves of SPM for METs with total immune score change below or above median. *P*-values were from log-rank test. **Figure S12.** Test association between survivals and relative percentage of M2-like macrophages of PBT/BRM pairs in Pan-MET dataset. (A) Kaplan-Meier (KM) curves of MFS for PBTs with relative percentage of M2-like macrophage below or above median. (B) KM curves of SPM for METs with relative percentage of M2-like macrophage below or above median. (C) KM curves of SPM for METs with relative percentage change of M2-like macrophage below or above median. P-values were from log-rank test.

**Additional file 3: Table S4.** Pathway enrichment analysis of differentially expressed genes in paired comparison of ER+ Brain METs versus matched PBTs.

**Additional file 4: Table S5.** Pathway enrichment analysis of differentially expressed genes in paired comparison of ER- Brain METs versus matched PBTs.

**Additional file 5: Table S6.** Pathway enrichment analysis of differentially expressed genes in comparison of ER+ ovarian METs versus matched PBTs.

**Additional file 6: Table S7.** Pathway enrichment analysis of differentially expressed genes in paired comparison of ER+ bone METs versus matched PBTs.

**Additional file 7: Table S8.** Test association between immune score and baseline clinical variables.

**Additional file 8: Table S9.** Test association between sTILs and baseline clinical variables.

## Data Availability

Data and code for all bioinformatic analyses are available on https://github.com/lizhu06/TILsComparison_PBTvsMET.

## Abbreviations

BOM: Metastatic breast tumor in bone
BRM: Metastatic breast tumor in brain
DE: Differential expression
ecRNA-seq: Exome-capture RNA sequencing
FFPE: Formalin fixed paraffin embedded
GI: Gastrointestinal tract
GIM: Metastatic breast tumor in gastrointestinal tract
GSVA: Gene set variation analysis
GTEx: Genotype-Tissue Expression
H&E: Hematoxylin & Eosin
HR: Hormone receptor
MET: Metastatic breast tumor
MFS: Metastasis-free-survival
mIF: Multiplexed immunofluorescence
OVM: Metastatic breast tumor in ovary
PBT: Primary breast tumor
SPM: Survival-post-metastasis
ssGSEA: Single-sample gene set enrichment analysis
sTIL: Stromal tumor infiltrating lymphocyte
TIL: Tumor infiltrating lymphocytes
TME: Tumor microenvironment
TNBC: Triple negative breast cancer

## References

[1] Noone AM, Howlader N, Krapcho M, Miller D, Brest A, Yu M, Ruhl J, Tatalovich Z, Mariotto A, Lewis DR, Chen HS, Feuer EJ, Cronin KA (eds). Cancer Statistics Review, 1975-2015, National Cancer Institute. Bethesda, MD, https://seer.cancer.gov/csr/1975_2015/. based on November 2017 SEER data submission, posted to the SEER web site, April 2018.

[2] Riihimäki Matias, Thomsen Hauke, Sundquist Kristina, Sundquist Jan, Hemminki Kari (2018). Clinical landscape of cancer metastases. Cancer Medicine.

[3] Dieci MV, Criscitiello C, Goubar A, Viale G, Conte P, Guarneri V, Ficarra G, Mathieu MC, Delaloge S, Curigliano G, Andre F (2014). Prognostic value of tumor-infiltrating lymphocytes on residual disease after primary chemotherapy for triple-negative breast cancer: a retrospective multicenter study. Ann Oncol.

[4] Perez EA, Ballman KV, Tenner KS, Thompson EA, Badve SS, Bailey H, Baehner FL (2016). Association of Stromal Tumor-Infiltrating Lymphocytes with Recurrence-Free Survival in the N9831 adjuvant trial in patients with early-stage HER2-positive breast Cancer. Jama Oncol.

[5] Zhang Mei, Lee Adrian V., Rosen Jeffrey M. (2017). The Cellular Origin and Evolution of Breast Cancer. Cold Spring Harbor Perspectives in Medicine.

[6] Brastianos PK, Carter SL, Santagata S, Cahill DP, Taylor-Weiner A, Jones RT, Van Allen EM, Lawrence MS, Horowitz PM, Cibulskis K, Ligon KL, Tabernero J, Seoane J, Martinez-Saez E, Curry WT, Dunn IF, Paek SH, Park SH, Mckenna A, Chevalier A, Rosenberg M, Barker FG, Gill CM, Van Hummelen P, Thorner AR, Johnson BE, Hoang MP, Choueiri TK, Signoretti S, Sougnez C, Rabin MS, Lin NU, Winer EP, Stemmer-Rachamimov A, Meyerson M, Garraway L, Gabriel S, Lander ES, Beroukhim R, Batchelor TT, Baselga J, Louis DN, Getz G, Hahn WC (2015). Genomic Characterization of Brain Metastases Reveals Branched Evolution and Potential Therapeutic Targets. Cancer Discov.

[7] Priedigkeit N, Hartmaier RJ, Chen Y, Vareslija D, Basudan A, Watters RJ, Thomas R, Leone JP, Lucas PC, Bhargava R, Hamilton RL, Chmielecki J, Puhalla SL, Davidson NE, Oesterreich S, Brufsky AM, Young L, Lee AV (2017). Intrinsic subtype switching and acquired ERBB2/HER2 amplifications and mutations in breast Cancer brain metastases. Jama Oncol.

[8] Priedigkeit N, Watters RJ, Lucas PC, Basudan A, Bhargava R, Horne W, Kolls JK, Fang Z, Rosenzweig MQ, Brufsky AM, Weiss KR, Oesterreich S, Lee AV. Exome-capture RNA sequencing of decade-old breast cancers and matched decalcified bone metastases. JCI Insight. 2017;2(17). 10.1172/jci.insight.95703. 10.1172/jci.insight.95703 PMC5621874 28878133

[9] Basudan Ahmed, Priedigkeit Nolan, Hartmaier Ryan J., Sokol Ethan S., Bahreini Amir, Watters Rebecca J., Boisen Michelle M., Bhargava Rohit, Weiss Kurt R., Karsten Maria M., Denkert Carsten, Blohmer Jens-Uwe, Leone Jose P., Hamilton Ronald L., Brufsky Adam M., Elishaev Esther, Lucas Peter C., Lee Adrian V., Oesterreich Steffi (2019). Frequent ESR1 and CDK Pathway Copy-Number Alterations in Metastatic Breast Cancer. Molecular Cancer Research.

[10] Yoshihara K, Shahmoradgoli M, Martinez E, Vegesna R, Kim H, Torres-Garcia W, Trevino V, Shen H, Laird PW, Levine DA, Carter SL, Getz G, Stemke-Hale K, Mills GB, Verhaak RG (2013). Inferring tumour purity and stromal and immune cell admixture from expression data. Nat Commun.

[11] Hanzelmann S, Castelo R, Guinney J (2013). GSVA: gene set variation analysis for microarray and RNA-seq data. BMC Bioinformatics.

[12] Newman AM, Liu CL, Green MR, Gentles AJ, Feng WG, Xu Y, Hoang CD, Diehn M, Alizadeh AA (2015). Robust enumeration of cell subsets from tissue expression profiles. Nat Methods.

[13] Li B, Severson E, Pignon JC, Zhao HQ, Li TW, Novak J, Jiang P, Shen H, Aster JC, Rodig S, Signoretti S, Liu JS, Liu XS (2016). Comprehensive analyses of tumor immunity: implications for cancer immunotherapy. Genome Biol.

[14] Salgado R, Denkert C, Demaria S, Sirtaine N, Klauschen F, Pruneri G, Wienert S, Van den Eynden G, Baehner FL, Penault-Llorca F, Perez EA, Thompson EA, Symmans WF, Richardson AL, Brock J, Criscitiello C, Bailey H, Ignatiadis M, Floris G, Sparano J, Kos Z, Nielsen T, Rimm DL, Allison KH, Reis-Filho JS, Loibl S, Sotiriou C, Viale G, Badve S, Adams S, Willard-Gallo K, Loi S, International TWG (2015). The evaluation of tumor-infiltrating lymphocytes (TILs) in breast cancer: recommendations by an international TILs working group 2014. Ann Oncol.

[15] Davoli Teresa, Uno Hajime, Wooten Eric C., Elledge Stephen J. (2017). Tumor aneuploidy correlates with markers of immune evasion and with reduced response to immunotherapy. Science.

[16] Tamborero D, Rubio-Perez C, Muinos F, Sabarinathan R, Piulats JM, Muntasell A, Dienstmann R, Lopez-Bigas N, Gonzalez-Perez A (2018). A pan-cancer landscape of interactions between solid tumors and infiltrating immune cell populations. Clin Cancer Res.

[17] Chung W, Eum HH, Lee HO, Lee KM, Lee HB, Kim KT, Ryu HS, Kim S, Lee JE, Park YH, Kan ZY, Han W, Park WY (2017). Single-cell RNA-seq enables comprehensive tumour and immune cell profiling in primary breast cancer. Nature Communications.

[18] Szekely B, Bossuyt V, Li XT, Baine M, Silber A, Sanft T, Hofstatter E, Mougalian S, Baghwagar S, Neumeister V, Pelekanou V, Hatzis C, Pusztai L. Immunological differences between primary and metastatic breast cancer. Cancer Res. 2018;78(4):2232-239. 10.1093/annonc/mdy399 30203045

[19] Cimino-Mathews A, Ye X, Meeker A, Argani P, Emens LA (2013). Metastatic triple-negative breast cancers at first relapse have fewer tumor-infiltrating lymphocytes than their matched primary breast tumors: a pilot study. Hum Pathol.

[20] Tawfik O, Kimler BF, Karnik T, Shehata P (2018). Clinicopathological correlation of PD-L1 expression in primary and metastatic breast cancer and infiltrating immune cells. Hum Pathol.

[21] Williams CB, Yeh ES, Soloff AC. Tumor-associated macrophages: unwitting accomplices in breast cancer malignancy. NPJ Breast Cancer. 2016;2. 10.1038/npjbcancer.2015.25. 10.1038/npjbcancer.2015.25 PMC4794275 26998515

[22] Qian BZ, Li JF, Zhang H, Kitamura T, Zhang JH, Campion LR, Kaiser EA, Snyder LA, Pollard JW (2011). CCL2 recruits inflammatory monocytes to facilitate breast-tumour metastasis. Nature.

[23] Linde N, Casanova-Acebes M, Sosa MS, Mortha A, Rahman A, Farias E, Harper K, Tardio E, Reyes Torres I, Jones J, Condeelis J, Merad M, Aguirre-Ghiso JA (2018). Macrophages orchestrate breast cancer early dissemination and metastasis. Nat Commun.

