# Supplementary material for: Metastatic breast cancers have reduced immune cell recruitment but harbor increased macrophages relative to their matched primary tumors
Source: J Immunother Cancer. 2019 Oct 18;7:265. doi: 10.1186/s40425-019-0755-1 (PMC6798422; doi:10.1186/s40425-019-0755-1)
Supplement: Supplementary file 1 — Additional file 1. Details of methods. [file 40425_2019_755_MOESM1_ESM.docx]

**Supplementary materials: Details of methods**

*Immune abundance quantification of samples in Pan-MET dataset*

Total immune score and tumor purity were calculated using R package ESTIMATE [1]. Abundance of each immune cell population were calculated by R package GSVA [2] based on two sets of immune gene signatures, Davoli signatures [3] and Tamborero signatures [4]. We also applied two deconvolution methods, CIBERSORT [5] and TIMER [6]. All four methods were tested on a single cell RNA-seq dataset of 11 breast cancer tumors [7].

*M2-like macrophages quantification of normal tissues*

RNA-seq data (TPM) from normal brain, breast, ovary and small intestine tissues were downloaded from The Genotype-Tissue Expression (GTEx) Project. We randomly selected 100 samples from each tissue and calculated the percentages of M2-like macrophages using CIBERSORT.

*Differential expression (DE) test and pathway enrichment analysis*

DE genes in ER+ BRMs versus PBTs were tested using R package DESeq2 [8]. Significantly up- or down-regulated genes were further used for pathway enrichment analyses. We obtained 2531 pathways, contributed by BioCarta, GO, KEGG, Reactome, containing 5–2000 genes, from Molecular Signature Database (MSigDB Version 5.1. Broad Institute, Cambridge, MA, USA). Fisher’s exact test was performed with false discovery rate (FDR) 0.05 as cutoff.

*Multiplex staining experiment of selective pairs in Pan-MET dataset*

FFPE tissue sections (5micron) were mounted on slides and deparaffinized. Briefly, tissues were subjected to cycles of antigen retrieval, blocking, primary antibody followed by secondary-HRP antibody. Separate Opal detection and signal amplification antibodies were used for each marker. The panel of markers used included CD8, CD20, CD68, Foxp3, PD-L1, pan-CK and DAPI. Imaging, analysis and quantification was performed using the Perkin Elmer Vectra platform and Inform software [9]. The list of antibodies with catalog numbers and dilutions used provided in supplementary Table S8.

*Evaluation of stromal tumor infiltrating lymphocytes (sTILs) in BRM-sTIL dataset samples.*

H&E stained sections were manually counted for percent sTILs using standard criteria developed by the international TILs working group [10]. sTILs were rounded to the nearest 5% increment. Only the stromal compartment within the borders of invasive tumor was evaluated. TILs in zones of necrosis, crushed artifacts, or normal tissue were excluded. Only mononuclear infiltrate was counted. Full tumor sections were preferentially examined over needle biopsies whenever possible; core biopsies were analyzed when full sections were unavailable. Each slide was independently reviewed by two study personnel (JLN and CL) to minimize inter-observer variability. When the sTILs differed by 10% or more, the study pathologist (AH) made the final determination. If multiple BRMs or PBTs were available for the same patient, average sTILs percentage was used for all comparisons.

**Reference**

1. Yoshihara K, Shahmoradgoli M, Martinez E, Vegesna R, Kim H, Torres-Garcia W, Trevino V, Shen H, Laird PW, Levine DA, Carter SL, Getz G, Stemke-Hale K, Mills GB, Verhaak RG (2013) Inferring tumour purity and stromal and immune cell admixture from expression data. Nat Commun 4:2612. doi:10.1038/ncomms3612

2. Hanzelmann S, Castelo R, Guinney J (2013) GSVA: gene set variation analysis for microarray and RNA-seq data. BMC Bioinformatics 14:7. doi:10.1186/1471-2105-14-7

3. Davoli T, Uno H, Wooten EC, Elledge SJ (2017) Tumor aneuploidy correlates with markers of immune evasion and with reduced response to immunotherapy. Science 355 (6322). doi:10.1126/science.aaf8399

4. Tamborero D, Rubio-Perez C, Muinos F, Sabarinathan R, Piulats JM, Muntasell A, Dienstmann R, Lopez-Bigas N, Gonzalez-Perez A (2018) A Pan-cancer Landscape of Interactions between Solid Tumors and Infiltrating Immune Cell Populations. Clin Cancer Res 24 (15):3717-3728. doi:10.1158/1078-0432.Ccr-17-3509

5. Newman AM, Liu CL, Green MR, Gentles AJ, Feng WG, Xu Y, Hoang CD, Diehn M, Alizadeh AA (2015) Robust enumeration of cell subsets from tissue expression profiles. Nat Methods 12 (5):453-+. doi:10.1038/Nmeth.3337

6. Li B, Severson E, Pignon JC, Zhao HQ, Li TW, Novak J, Jiang P, Shen H, Aster JC, Rodig S, Signoretti S, Liu JS, Liu XS (2016) Comprehensive analyses of tumor immunity: implications for cancer immunotherapy. Genome Biol 17. doi:ARTN 174

10.1186/s13059-016-1028-7

7. Chung W, Eum HH, Lee HO, Lee KM, Lee HB, Kim KT, Ryu HS, Kim S, Lee JE, Park YH, Kan ZY, Han W, Park WY (2017) Single-cell RNA-seq enables comprehensive tumour and immune cell profiling in primary breast cancer. Nature Communications 8. doi:ARTN 15081

10.1038/ncomms15081

8. Love MI, Huber W, Anders S (2014) Moderated estimation of fold change and dispersion for RNA-seq data with DESeq2. Genome Biol 15 (12):550. doi:10.1186/s13059-014-0550-8

9. Stack EC, Wang C, Roman KA, Hoyt CC (2014) Multiplexed immunohistochemistry, imaging, and quantitation: a review, with an assessment of Tyramide signal amplification, multispectral imaging and multiplex analysis. Methods 70 (1):46-58. doi:10.1016/j.ymeth.2014.08.016

10. Salgado R, Denkert C, Demaria S, Sirtaine N, Klauschen F, Pruneri G, Wienert S, Van den Eynden G, Baehner FL, Penault-Llorca F, Perez EA, Thompson EA, Symmans WF, Richardson AL, Brock J, Criscitiello C, Bailey H, Ignatiadis M, Floris G, Sparano J, Kos Z, Nielsen T, Rimm DL, Allison KH, Reis-Filho JS, Loibl S, Sotiriou C, Viale G, Badve S, Adams S, Willard-Gallo K, Loi S, International TWG (2015) The evaluation of tumor-infiltrating lymphocytes (TILs) in breast cancer: recommendations by an International TILs Working Group 2014. Ann Oncol 26 (2):259-271. doi:10.1093/annonc/mdu450
