# Supplementary material for: Metastatic breast cancers have reduced immune cell recruitment but harbor increased macrophages relative to their matched primary tumors
Source: J Immunother Cancer. 2019 Oct 18;7:265. doi: 10.1186/s40425-019-0755-1 (PMC6798422; doi:10.1186/s40425-019-0755-1)
Supplement: Supplementary file 2 — Additional file 2: Table S1. Clinical information of samples in Pan-MET dataset. Table S2. Clinical information of samples in BRM-sTIL dataset. Table S3. 15 pairs of PBT/BRMs overlap between the Pan-MET and BRM-sTIL. Table S10. Detailed list of antibodies and dilutions used for multispectral immunofluorescence staining of slides as shown in Fig. 3. Figure S1. Comparison of immune abundance in metastatic breast tumors (METs) and primary breast tumors (PBTs) grouped by HR/HER2 subtypes. (A) Total Immune score in Pan-MET dataset, together with the paired changes (MET-PBT). (B) sTILs percentages of PBT/BRM pairs in BRM-sTIL dataset, together with the paired changes (MET-PBT). ****p < 0.0001, ***p < 0.001, **p < 0.01, *p < 0.05 from two-sided Wilcoxon signed rank test. Figure S2. Comparison of immune abundance in METs and PBTs, treating multiple METs matched to the same PBT as MET in different pairs. (A) Total immune score in PBT/MET pairs in Pan-MET dataset. (B) Total immune score grouped by MET sites. (C) Total immune score in Pan-MET dataset grouped by HR/HER2 subtypes. ****p < 0.0001, ***p < 0.001, **p < 0.01, *p < 0.05 from two-sided Wilcoxon signed rank test in (A-C) and correlation test in (D). Figure S3. Expression (log2(TPM + 1)) of CD274 (PD-L1), PDCD1 (PD-1), and CTAL4 in PBT and MET. Two-sided Wilcoxon signed rank test was used to compare PBT and MET. Spearman’s correlation with immune score change was calculated and tested using correlation test. ****p < 0.0001, ***p < 0.001, **p < 0.01, *p < 0.05. Figure S4. Correlation between GSVA scores of Davoli and Tamborero signatures for PBT/MET pairs in Pan-MET dataset. Figure S5. Correlation between immune abundance estimated from RNA-seq data and cell count/proportion (relative to total immune cell count) in single cell RNA-seq dataset. (A-B) GSVA score of (A) Davoli and (B) Tamborero signatures. (C) Percentage relative to total immune level estimated by CIBERSORT. (D) Immune abundance estimated by TIMER. White in [file 40425_2019_755_MOESM2_ESM.pptx]

## Slide 1
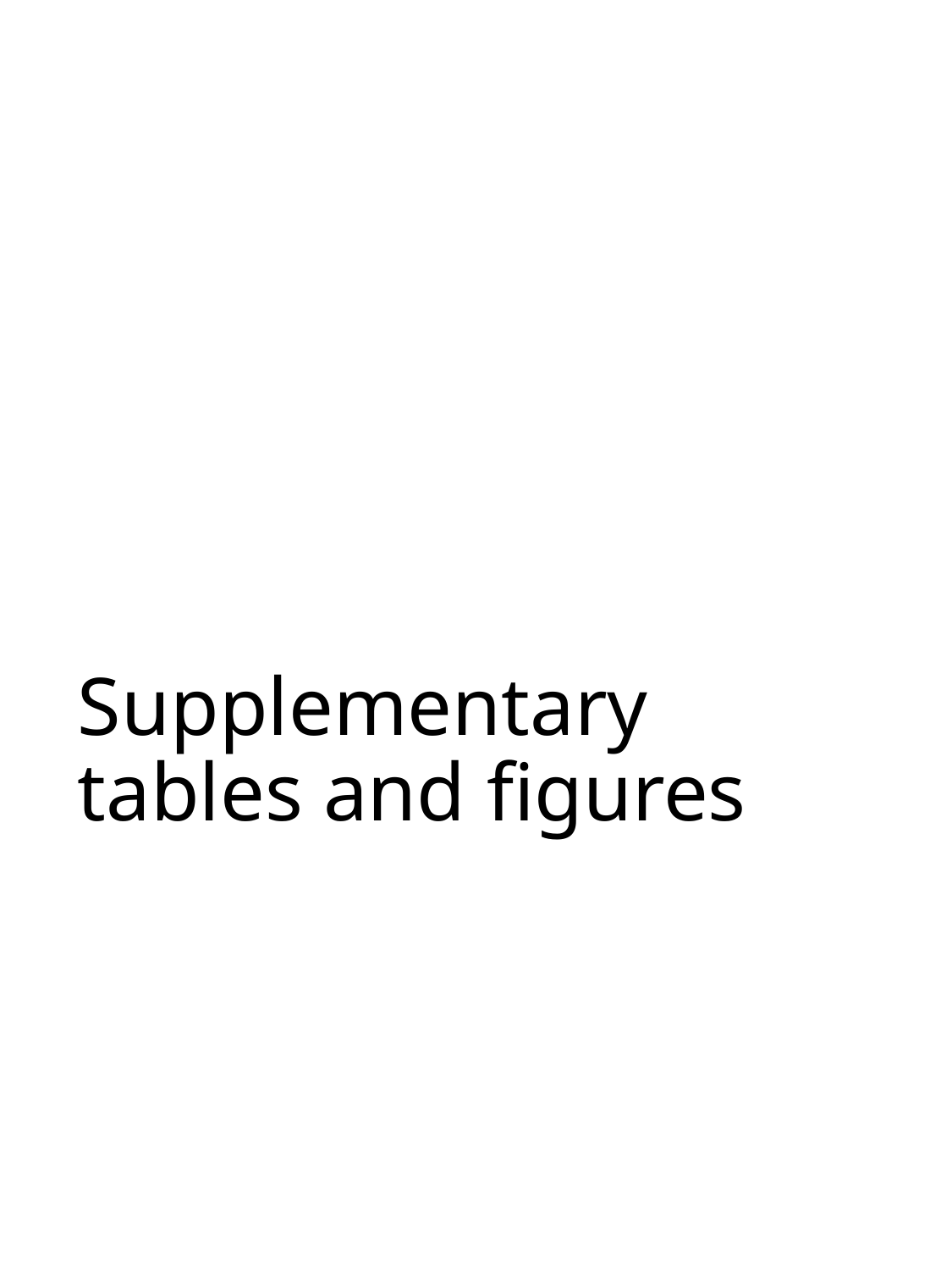

# Supplementary tables and figures

## Slide 2
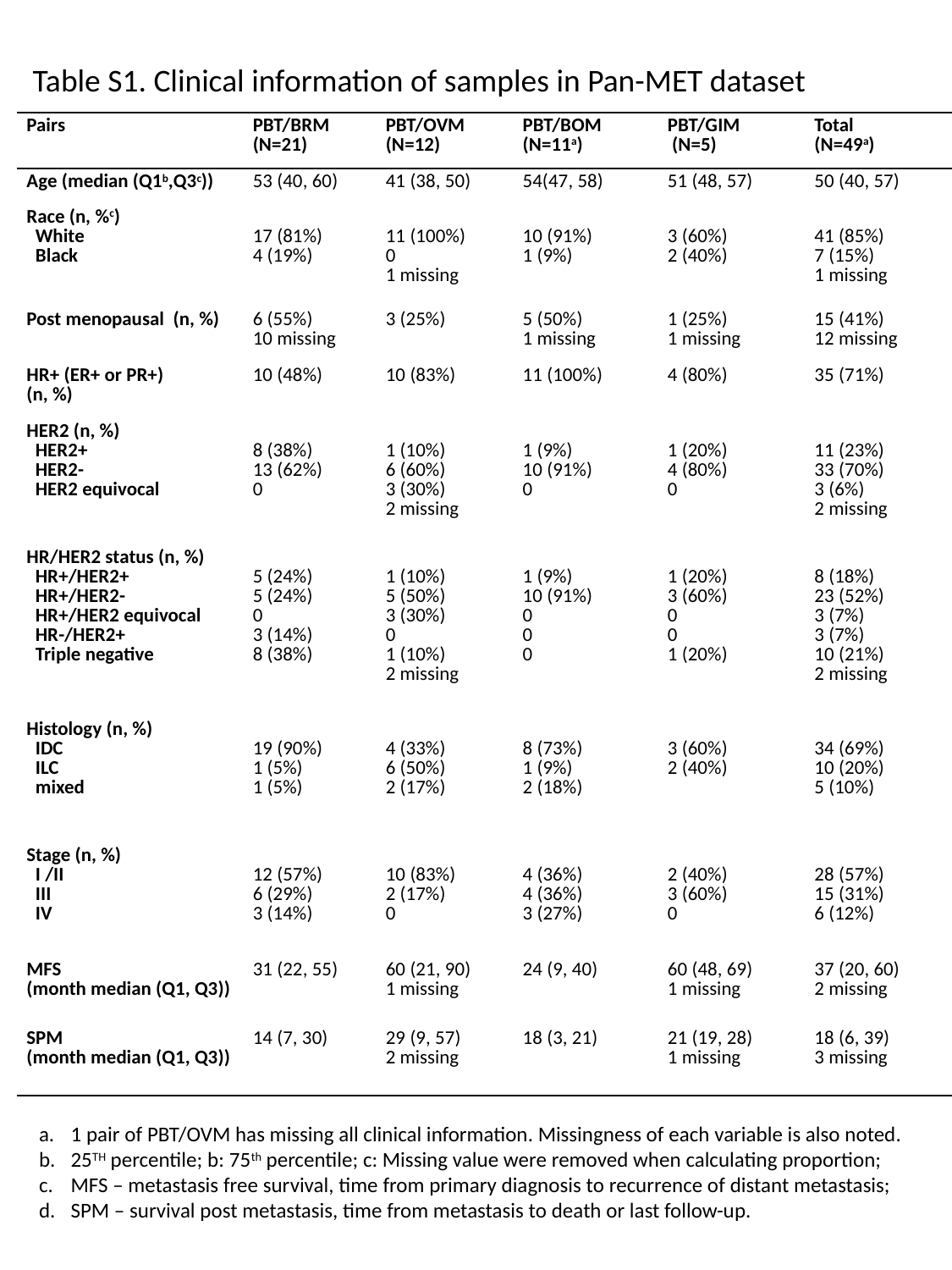

Table S1. Clinical information of samples in Pan-MET dataset
| Pairs | PBT/BRM (N=21) | PBT/OVM (N=12) | PBT/BOM (N=11a) | PBT/GIM (N=5) | Total (N=49a) |
| --- | --- | --- | --- | --- | --- |
| Age (median (Q1b,Q3c)) | 53 (40, 60) | 41 (38, 50) | 54(47, 58) | 51 (48, 57) | 50 (40, 57) |
| Race (n, %c) White Black | 17 (81%) 4 (19%) | 11 (100%) 0 1 missing | 10 (91%) 1 (9%) | 3 (60%) 2 (40%) | 41 (85%) 7 (15%) 1 missing |
| Post menopausal (n, %) | 6 (55%) 10 missing | 3 (25%) | 5 (50%) 1 missing | 1 (25%) 1 missing | 15 (41%) 12 missing |
| HR+ (ER+ or PR+) (n, %) | 10 (48%) | 10 (83%) | 11 (100%) | 4 (80%) | 35 (71%) |
| HER2 (n, %) HER2+ HER2- HER2 equivocal | 8 (38%) 13 (62%) 0 | 1 (10%) 6 (60%) 3 (30%) 2 missing | 1 (9%) 10 (91%) 0 | 1 (20%) 4 (80%) 0 | 11 (23%) 33 (70%) 3 (6%) 2 missing |
| HR/HER2 status (n, %) HR+/HER2+ HR+/HER2- HR+/HER2 equivocal HR-/HER2+ Triple negative | 5 (24%) 5 (24%) 0 3 (14%) 8 (38%) | 1 (10%) 5 (50%) 3 (30%) 0 1 (10%) 2 missing | 1 (9%) 10 (91%) 0 0 0 | 1 (20%) 3 (60%) 0 0 1 (20%) | 8 (18%) 23 (52%) 3 (7%) 3 (7%) 10 (21%) 2 missing |
| Histology (n, %) IDC ILC mixed | 19 (90%) 1 (5%) 1 (5%) | 4 (33%) 6 (50%) 2 (17%) | 8 (73%) 1 (9%) 2 (18%) | 3 (60%) 2 (40%) | 34 (69%) 10 (20%) 5 (10%) |
| Stage (n, %) I /II III IV | 12 (57%) 6 (29%) 3 (14%) | 10 (83%) 2 (17%) 0 | 4 (36%) 4 (36%) 3 (27%) | 2 (40%) 3 (60%) 0 | 28 (57%) 15 (31%) 6 (12%) |
| MFS (month median (Q1, Q3)) | 31 (22, 55) | 60 (21, 90) 1 missing | 24 (9, 40) | 60 (48, 69) 1 missing | 37 (20, 60) 2 missing |
| SPM (month median (Q1, Q3)) | 14 (7, 30) | 29 (9, 57) 2 missing | 18 (3, 21) | 21 (19, 28) 1 missing | 18 (6, 39) 3 missing |
1 pair of PBT/OVM has missing all clinical information. Missingness of each variable is also noted.
25TH percentile; b: 75th percentile; c: Missing value were removed when calculating proportion;
MFS – metastasis free survival, time from primary diagnosis to recurrence of distant metastasis;
SPM – survival post metastasis, time from metastasis to death or last follow-up.

## Slide 3
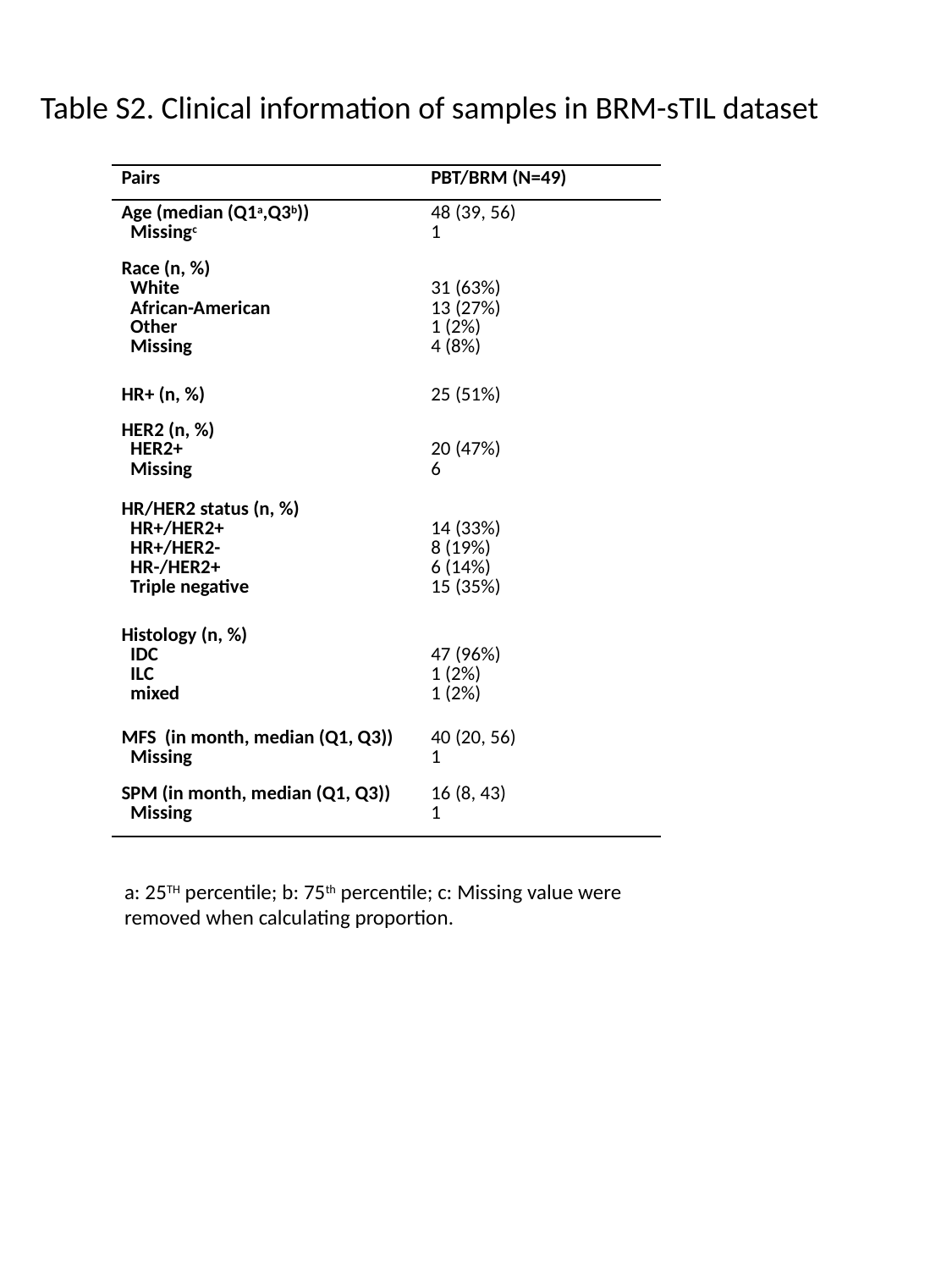

Table S2. Clinical information of samples in BRM-sTIL dataset
| Pairs | PBT/BRM (N=49) |
| --- | --- |
| Age (median (Q1a,Q3b)) Missingc | 48 (39, 56) 1 |
| Race (n, %) White African-American Other Missing | 31 (63%) 13 (27%) 1 (2%) 4 (8%) |
| HR+ (n, %) | 25 (51%) |
| HER2 (n, %) HER2+ Missing | 20 (47%) 6 |
| HR/HER2 status (n, %) HR+/HER2+ HR+/HER2- HR-/HER2+ Triple negative | 14 (33%) 8 (19%) 6 (14%) 15 (35%) |
| Histology (n, %) IDC ILC mixed | 47 (96%) 1 (2%) 1 (2%) |
| MFS (in month, median (Q1, Q3)) Missing | 40 (20, 56) 1 |
| SPM (in month, median (Q1, Q3)) Missing | 16 (8, 43) 1 |
a: 25TH percentile; b: 75th percentile; c: Missing value were removed when calculating proportion.

## Slide 4
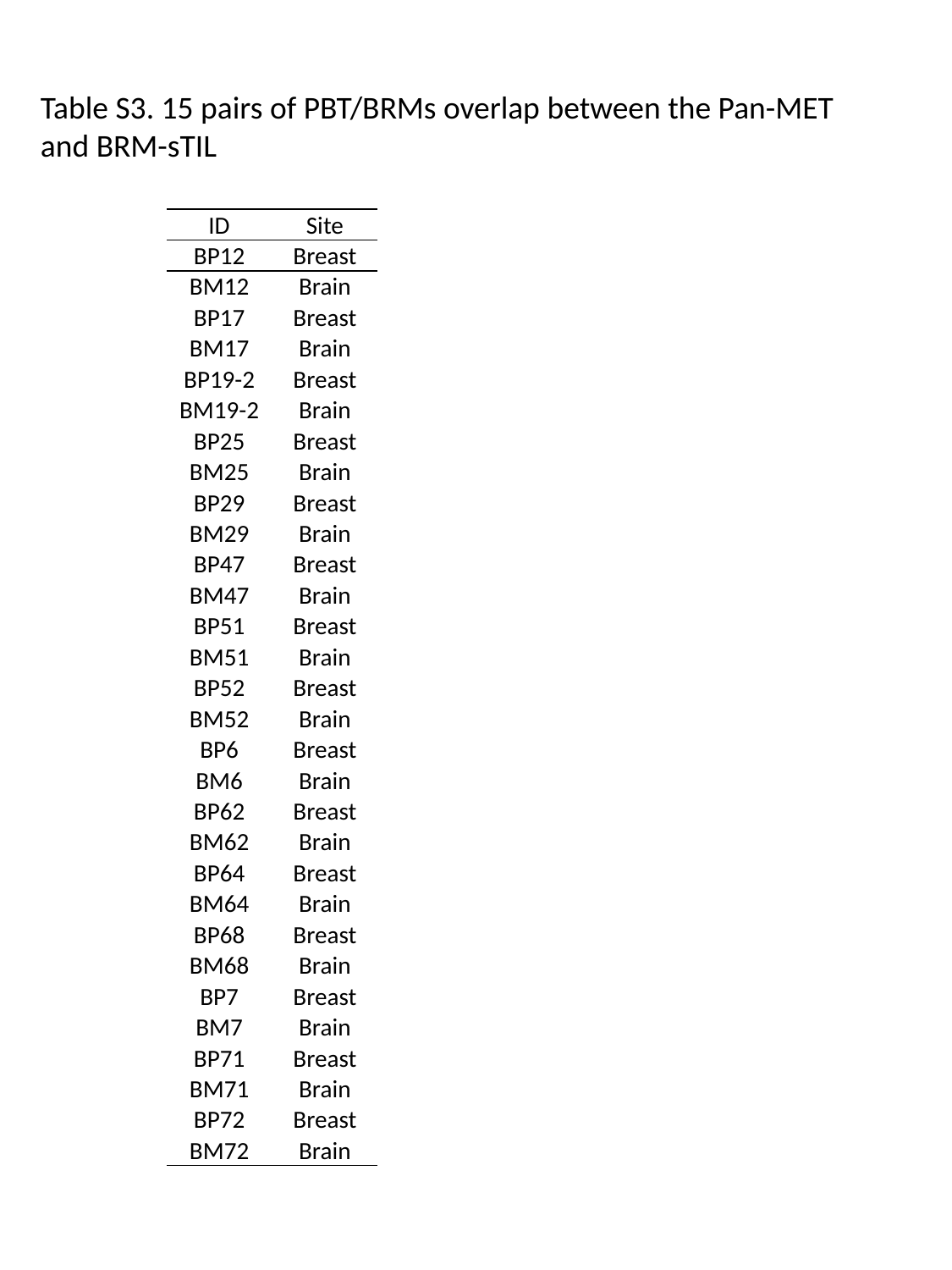

Table S3. 15 pairs of PBT/BRMs overlap between the Pan-MET and BRM-sTIL
| ID | Site |
| --- | --- |
| BP12 | Breast |
| BM12 | Brain |
| BP17 | Breast |
| BM17 | Brain |
| BP19-2 | Breast |
| BM19-2 | Brain |
| BP25 | Breast |
| BM25 | Brain |
| BP29 | Breast |
| BM29 | Brain |
| BP47 | Breast |
| BM47 | Brain |
| BP51 | Breast |
| BM51 | Brain |
| BP52 | Breast |
| BM52 | Brain |
| BP6 | Breast |
| BM6 | Brain |
| BP62 | Breast |
| BM62 | Brain |
| BP64 | Breast |
| BM64 | Brain |
| BP68 | Breast |
| BM68 | Brain |
| BP7 | Breast |
| BM7 | Brain |
| BP71 | Breast |
| BM71 | Brain |
| BP72 | Breast |
| BM72 | Brain |

## Slide 5
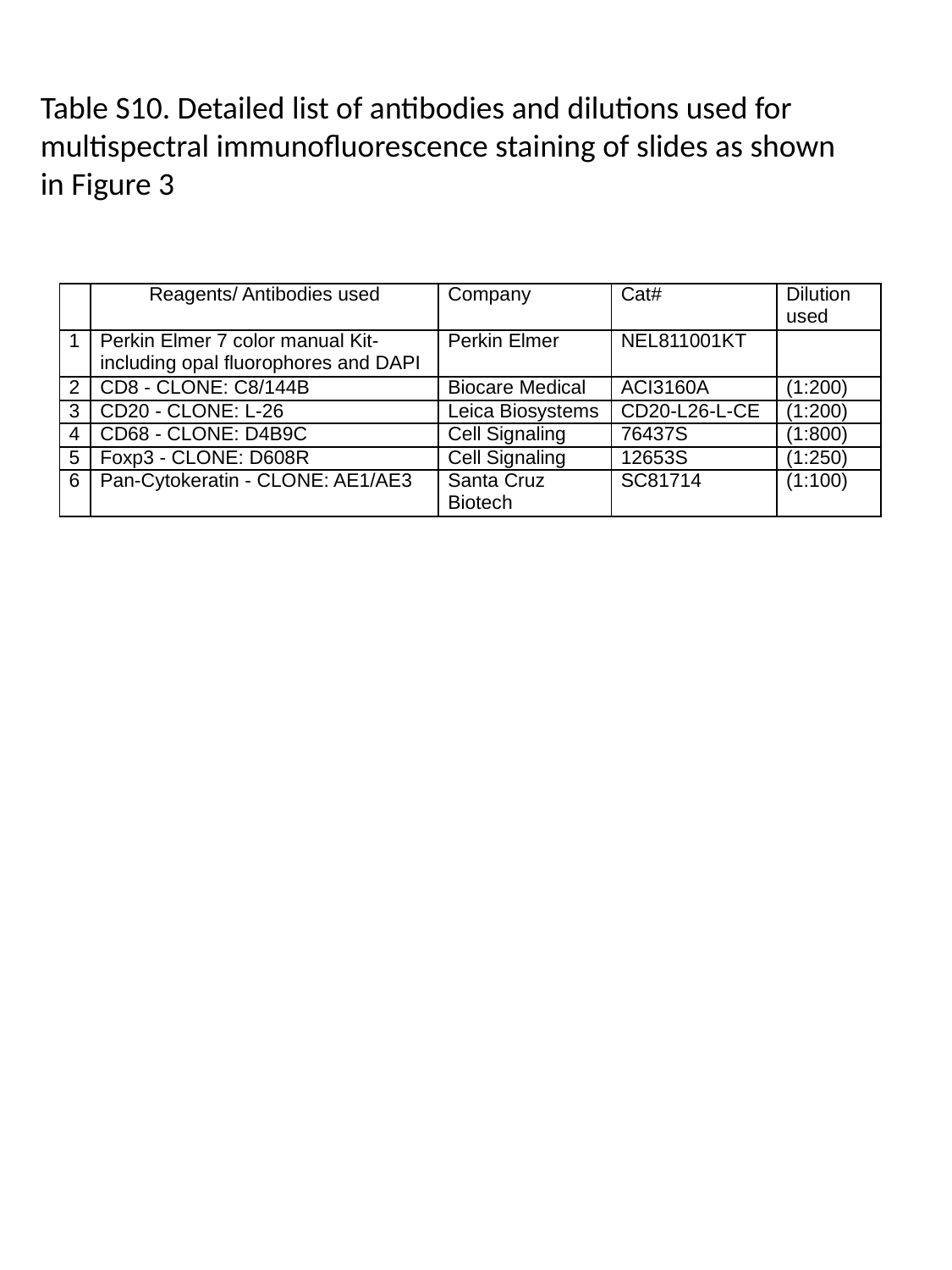

Table S10. Detailed list of antibodies and dilutions used for multispectral immunofluorescence staining of slides as shown in Figure 3
| | Reagents/ Antibodies used | Company | Cat# | Dilution used |
| --- | --- | --- | --- | --- |
| 1 | Perkin Elmer 7 color manual Kit- including opal fluorophores and DAPI | Perkin Elmer | NEL811001KT | |
| 2 | CD8 - CLONE: C8/144B | Biocare Medical | ACI3160A | (1:200) |
| 3 | CD20 - CLONE: L-26 | Leica Biosystems | CD20-L26-L-CE | (1:200) |
| 4 | CD68 - CLONE: D4B9C | Cell Signaling | 76437S | (1:800) |
| 5 | Foxp3 - CLONE: D608R | Cell Signaling | 12653S | (1:250) |
| 6 | Pan-Cytokeratin - CLONE: AE1/AE3 | Santa Cruz Biotech | SC81714 | (1:100) |

## Slide 6
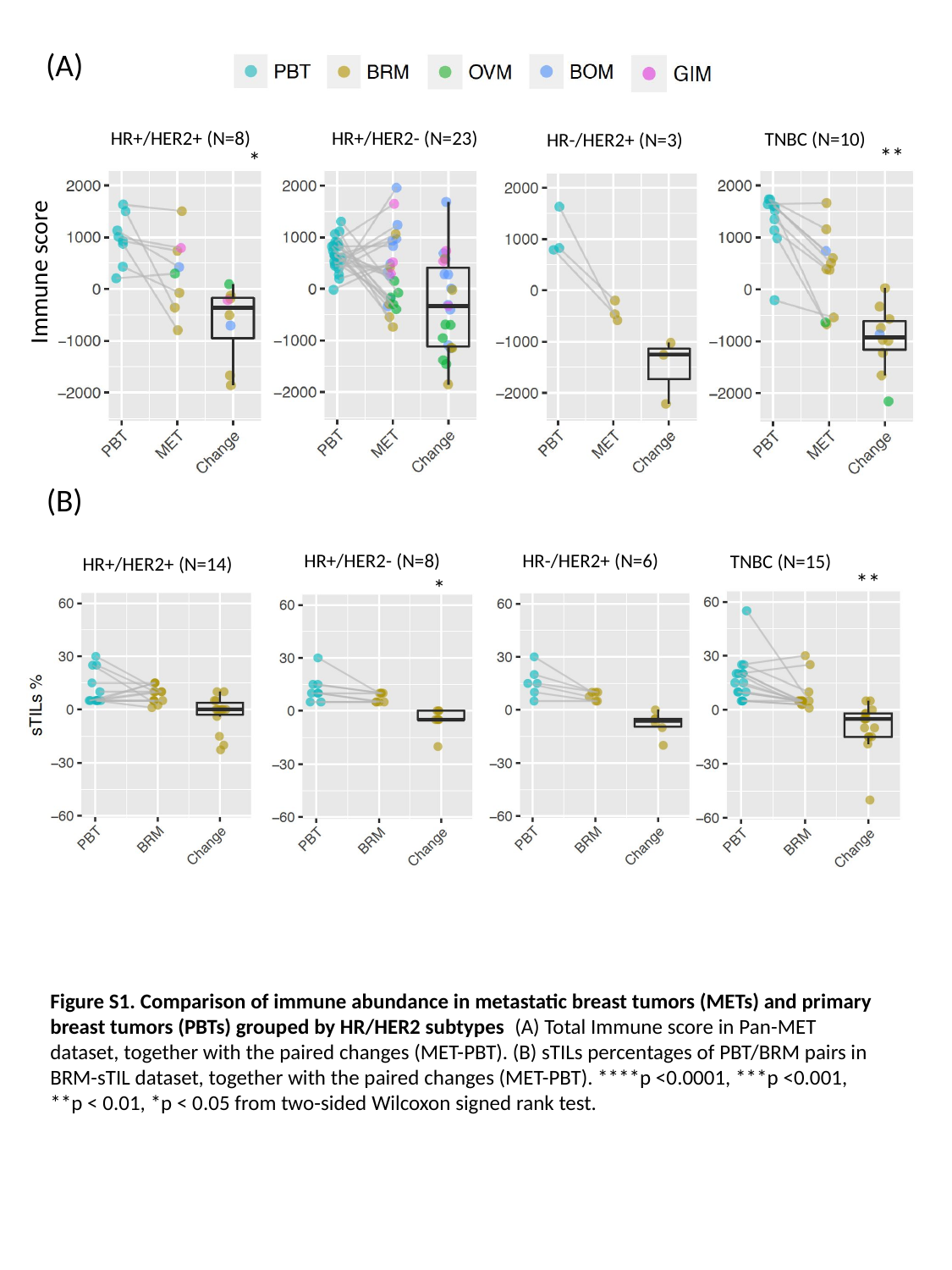

(A)
HR+/HER2+ (N=8)
HR+/HER2- (N=23)
TNBC (N=10)
HR-/HER2+ (N=3)
**
*
Immune score
(B)
HR-/HER2+ (N=6)
HR+/HER2- (N=8)
TNBC (N=15)
HR+/HER2+ (N=14)
**
*
Figure S1. Comparison of immune abundance in metastatic breast tumors (METs) and primary breast tumors (PBTs) grouped by HR/HER2 subtypes (A) Total Immune score in Pan-MET dataset, together with the paired changes (MET-PBT). (B) sTILs percentages of PBT/BRM pairs in BRM-sTIL dataset, together with the paired changes (MET-PBT). ****p <0.0001, ***p <0.001, **p < 0.01, *p < 0.05 from two-sided Wilcoxon signed rank test.

## Slide 7
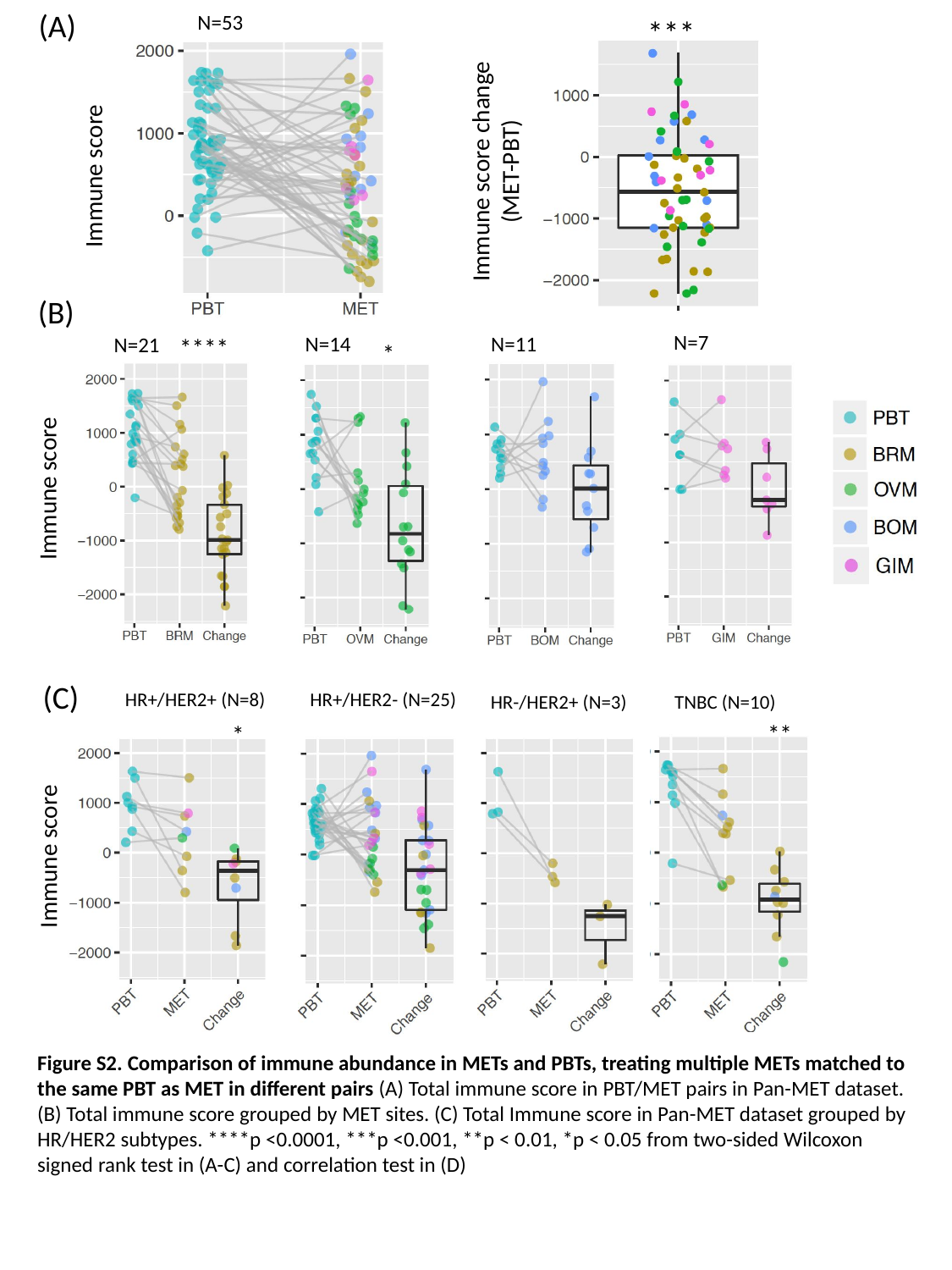

(A)
N=53
***
Immune score change
(MET-PBT)
Immune score
(B)
N=7
N=11
N=14
N=21
****
*
Immune score
(C)
HR+/HER2- (N=25)
HR+/HER2+ (N=8)
TNBC (N=10)
HR-/HER2+ (N=3)
**
*
Immune score
Figure S2. Comparison of immune abundance in METs and PBTs, treating multiple METs matched to the same PBT as MET in different pairs (A) Total immune score in PBT/MET pairs in Pan-MET dataset. (B) Total immune score grouped by MET sites. (C) Total Immune score in Pan-MET dataset grouped by HR/HER2 subtypes. ****p <0.0001, ***p <0.001, **p < 0.01, *p < 0.05 from two-sided Wilcoxon signed rank test in (A-C) and correlation test in (D)

## Slide 8
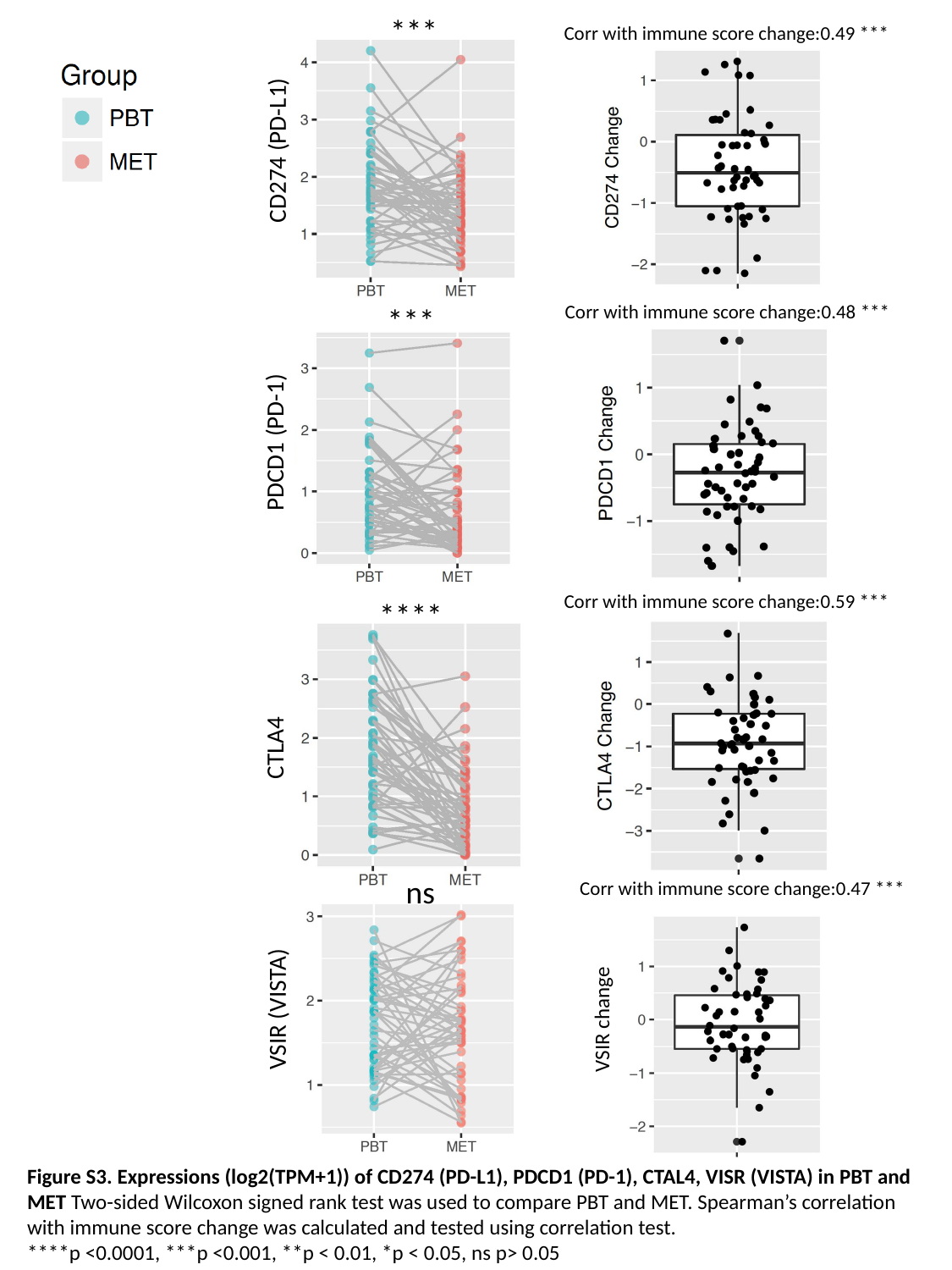

***
Corr with immune score change:0.49 ***
CD274 (PD-L1)
Corr with immune score change:0.48 ***
***
PDCD1 (PD-1)
Corr with immune score change:0.59 ***
****
CTLA4
ns
Corr with immune score change:0.47 ***
VSIR (VISTA)
VSIR change
Figure S3. Expressions (log2(TPM+1)) of CD274 (PD-L1), PDCD1 (PD-1), CTAL4, VISR (VISTA) in PBT and MET Two-sided Wilcoxon signed rank test was used to compare PBT and MET. Spearman’s correlation with immune score change was calculated and tested using correlation test.
****p <0.0001, ***p <0.001, **p < 0.01, *p < 0.05, ns p> 0.05

## Slide 9
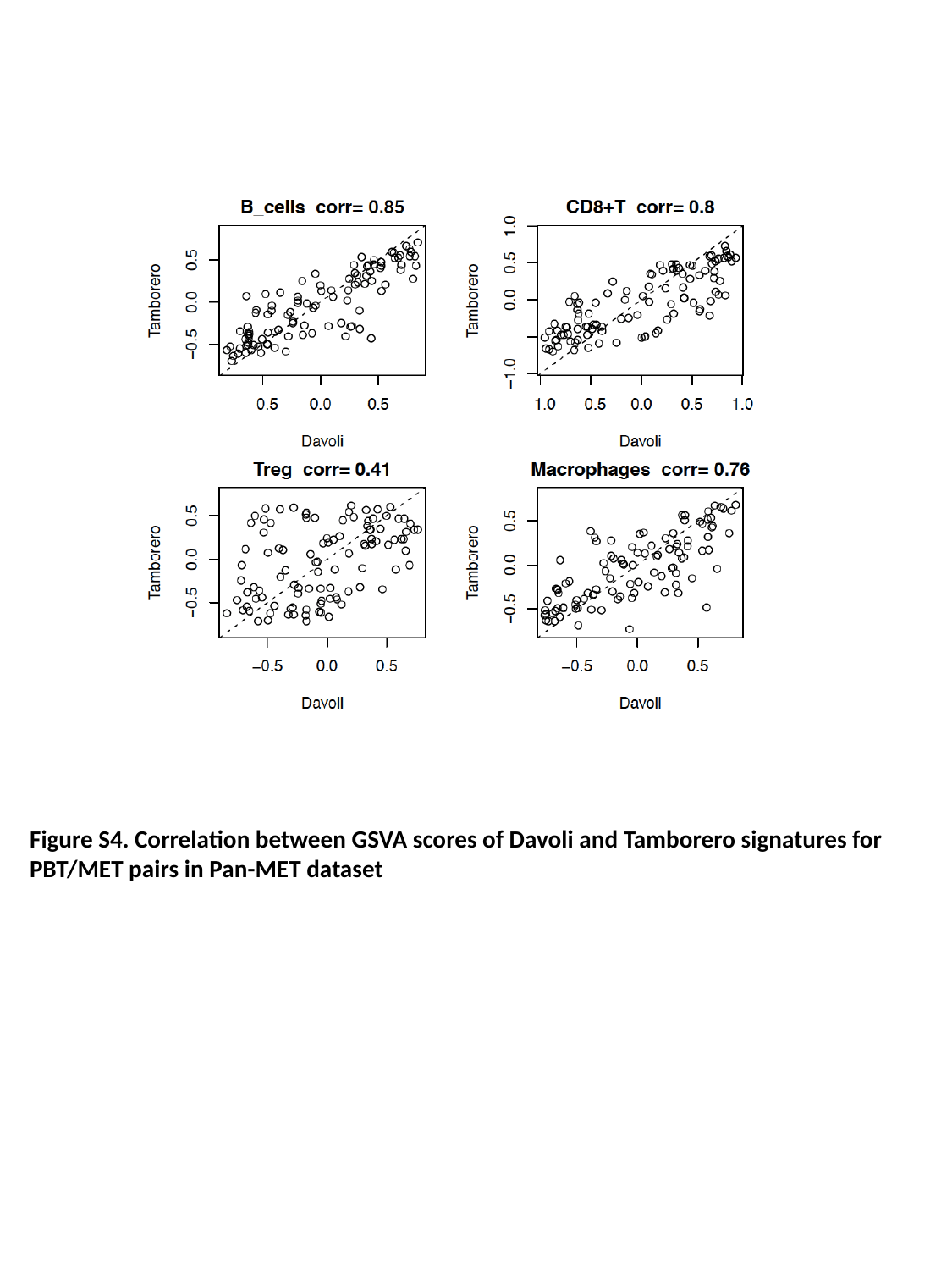

Figure S4. Correlation between GSVA scores of Davoli and Tamborero signatures for PBT/MET pairs in Pan-MET dataset

## Slide 10
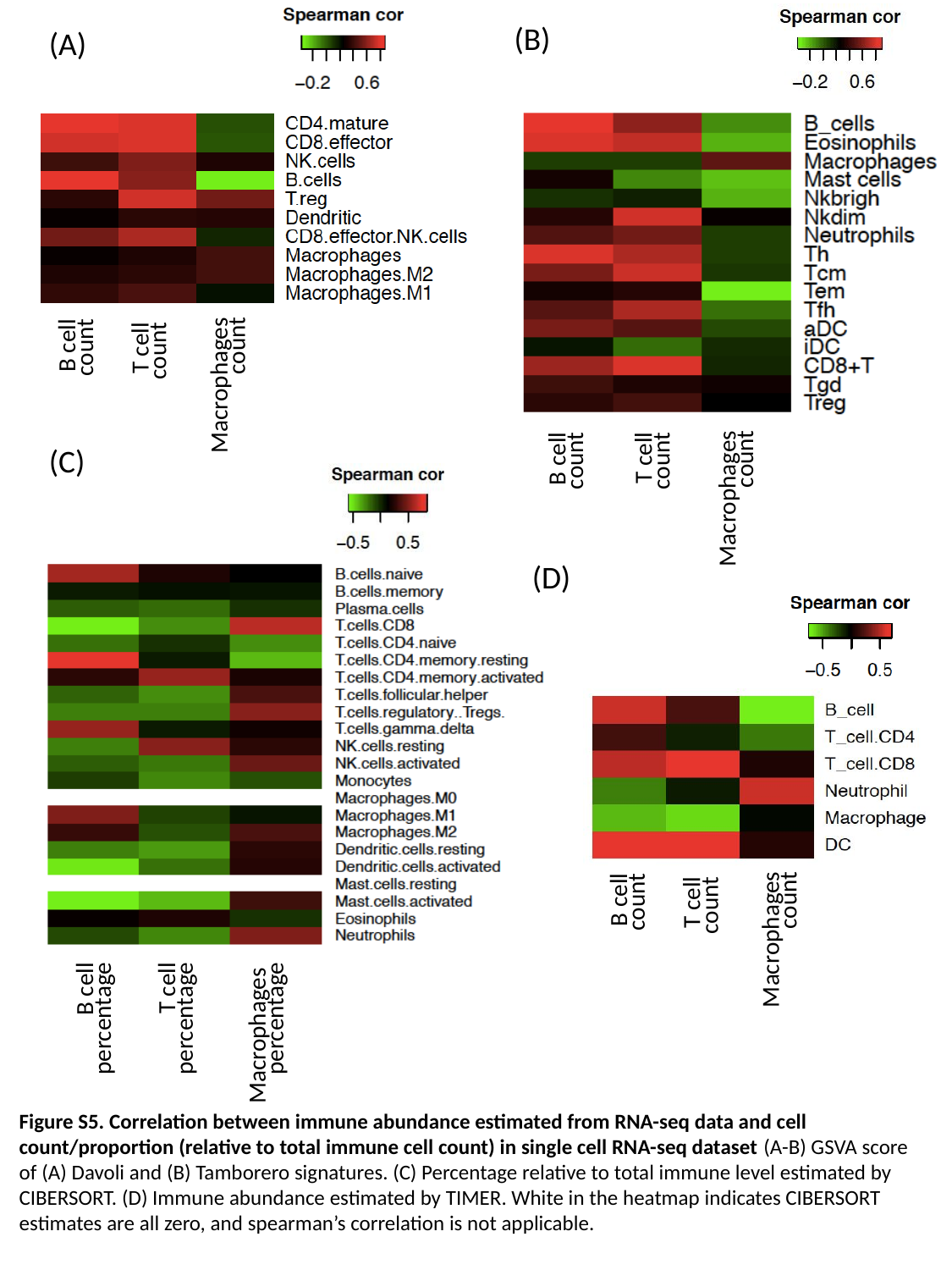

(B)
(A)
B cell count
T cell count
Macrophages count
(C)
B cell count
T cell count
Macrophages count
(D)
B cell count
T cell count
Macrophages count
T cell percentage
B cell percentage
Macrophages percentage
Figure S5. Correlation between immune abundance estimated from RNA-seq data and cell count/proportion (relative to total immune cell count) in single cell RNA-seq dataset (A-B) GSVA score of (A) Davoli and (B) Tamborero signatures. (C) Percentage relative to total immune level estimated by CIBERSORT. (D) Immune abundance estimated by TIMER. White in the heatmap indicates CIBERSORT estimates are all zero, and spearman’s correlation is not applicable.

## Slide 11
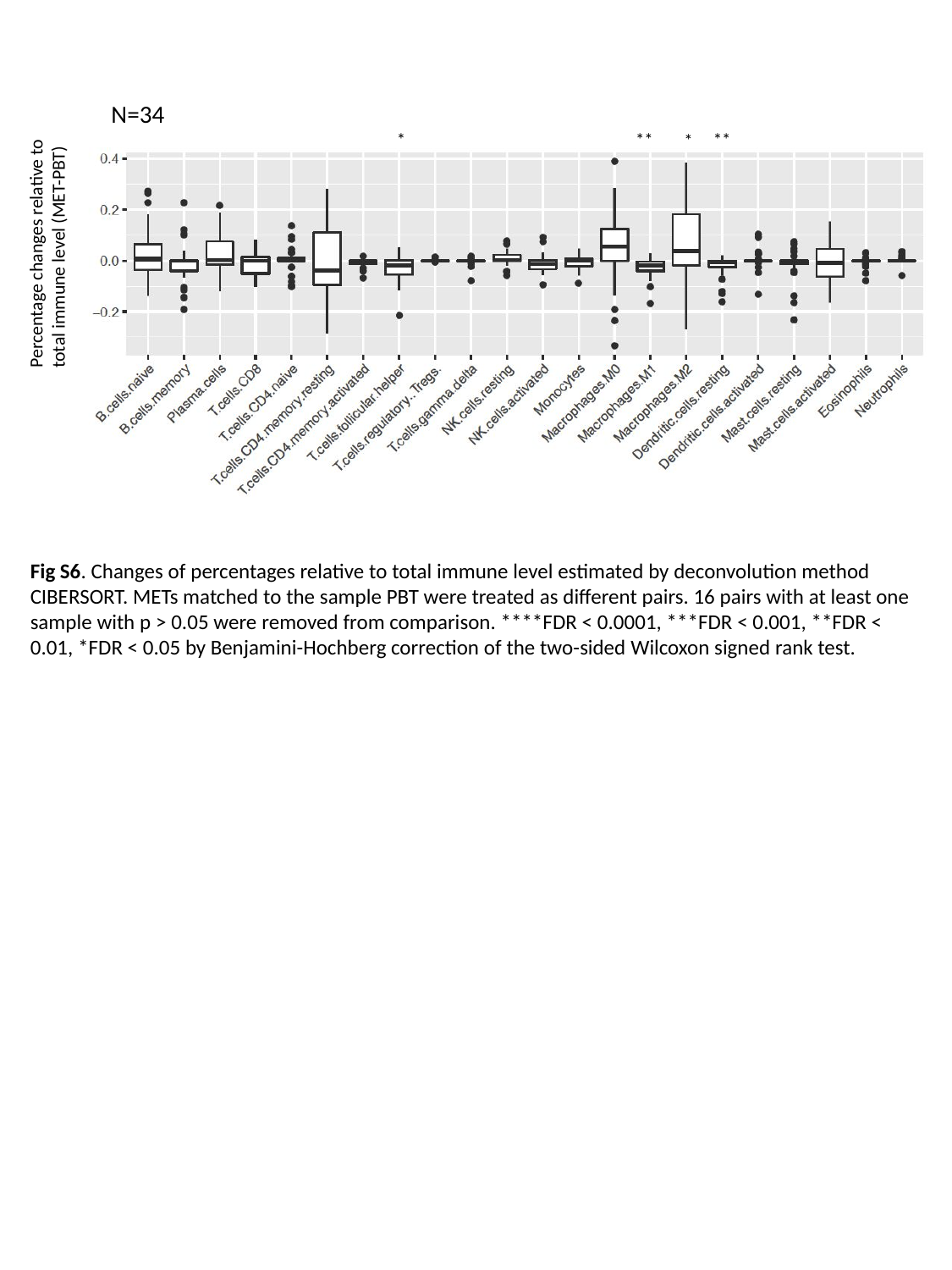

N=34
**
*
**
*
Percentage changes relative to
total immune level (MET-PBT)
Fig S6. Changes of percentages relative to total immune level estimated by deconvolution method CIBERSORT. METs matched to the sample PBT were treated as different pairs. 16 pairs with at least one sample with p > 0.05 were removed from comparison. ****FDR < 0.0001, ***FDR < 0.001, **FDR < 0.01, *FDR < 0.05 by Benjamini-Hochberg correction of the two-sided Wilcoxon signed rank test.

## Slide 12
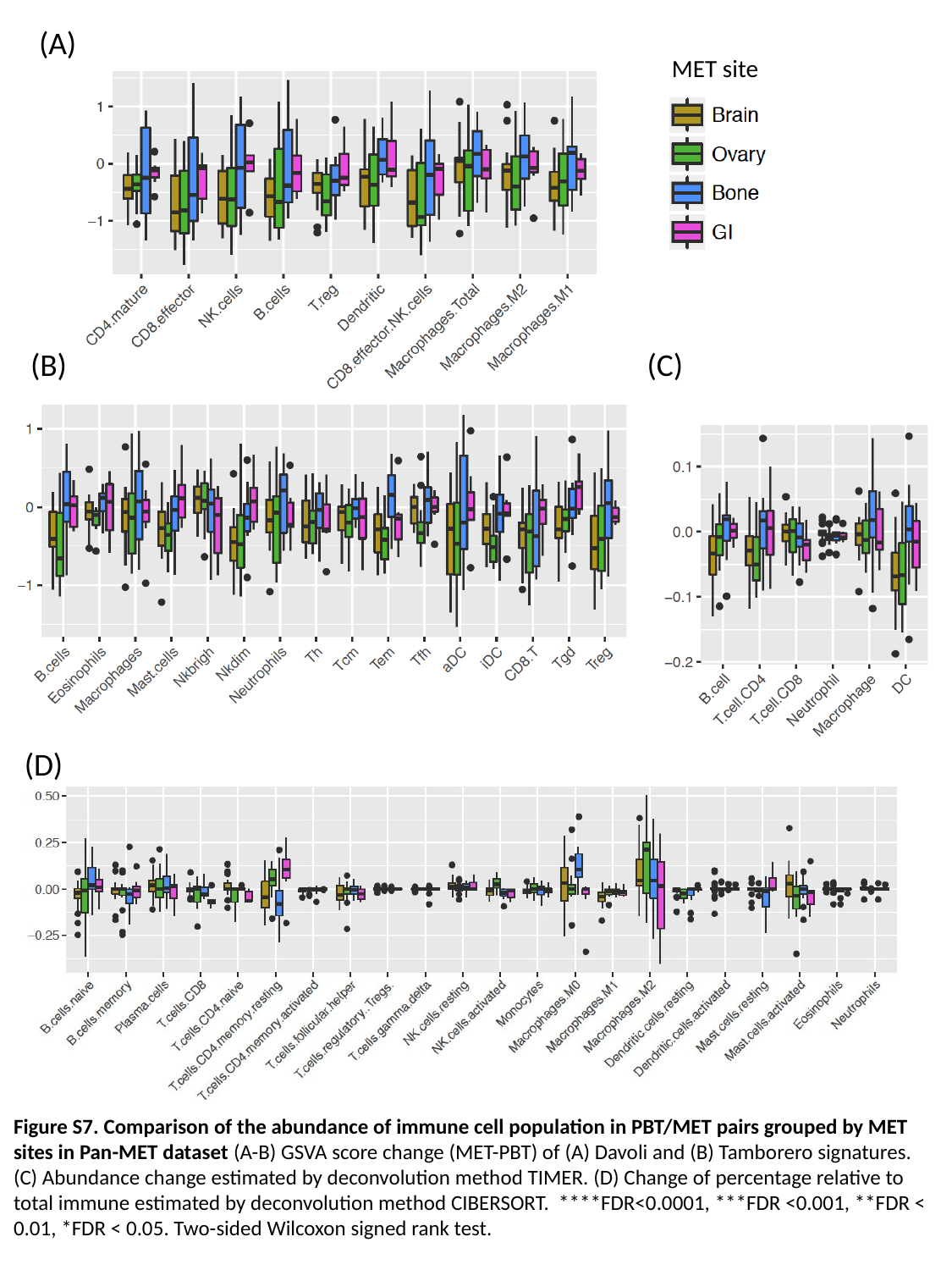

(A)
MET site
(B)
(C)
(D)
Figure S7. Comparison of the abundance of immune cell population in PBT/MET pairs grouped by MET sites in Pan-MET dataset (A-B) GSVA score change (MET-PBT) of (A) Davoli and (B) Tamborero signatures. (C) Abundance change estimated by deconvolution method TIMER. (D) Change of percentage relative to total immune estimated by deconvolution method CIBERSORT. ****FDR<0.0001, ***FDR <0.001, **FDR < 0.01, *FDR < 0.05. Two-sided Wilcoxon signed rank test.

## Slide 13
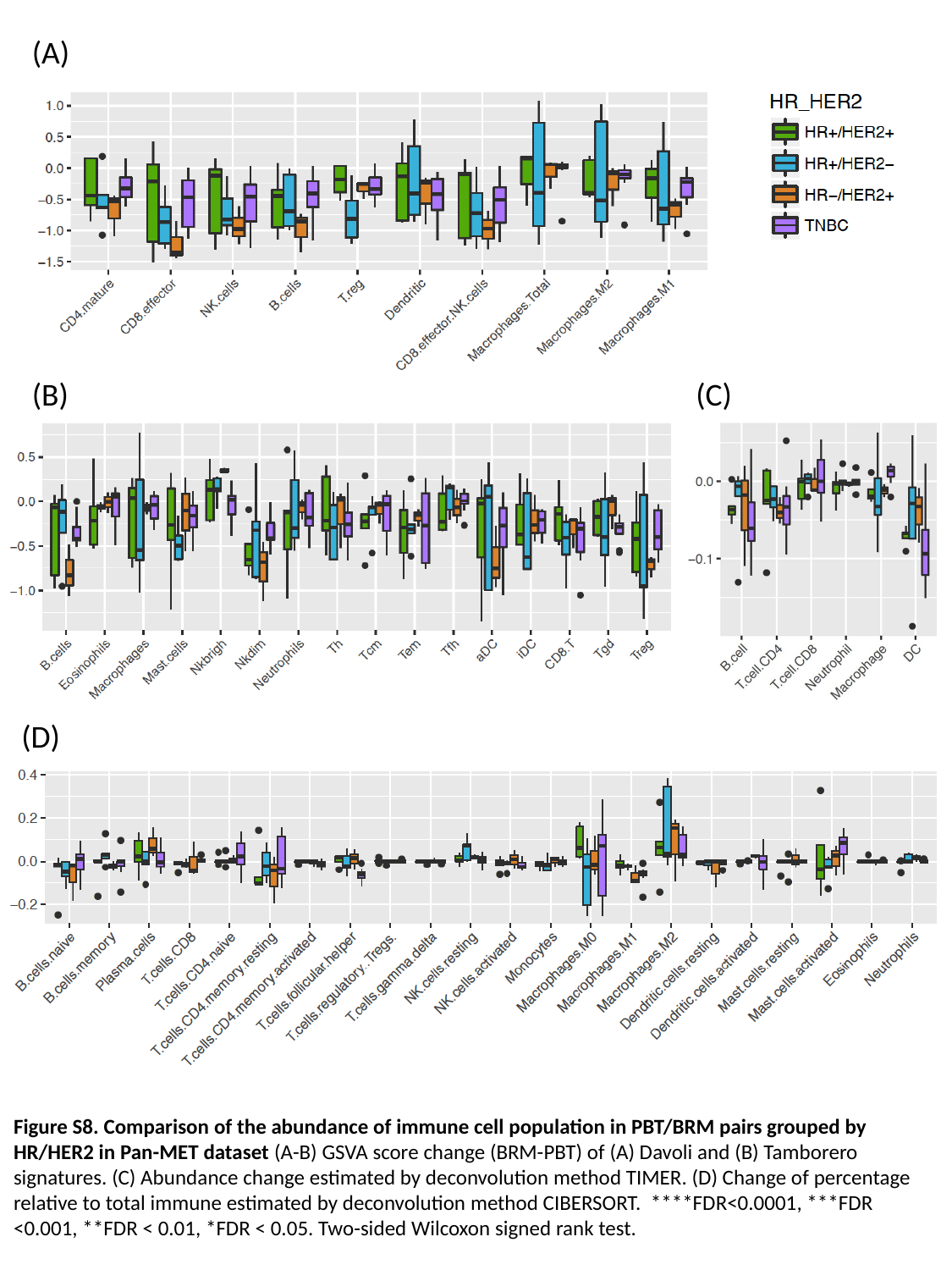

(A)
(B)
(C)
(D)
Figure S8. Comparison of the abundance of immune cell population in PBT/BRM pairs grouped by HR/HER2 in Pan-MET dataset (A-B) GSVA score change (BRM-PBT) of (A) Davoli and (B) Tamborero signatures. (C) Abundance change estimated by deconvolution method TIMER. (D) Change of percentage relative to total immune estimated by deconvolution method CIBERSORT. ****FDR<0.0001, ***FDR <0.001, **FDR < 0.01, *FDR < 0.05. Two-sided Wilcoxon signed rank test.

## Slide 14
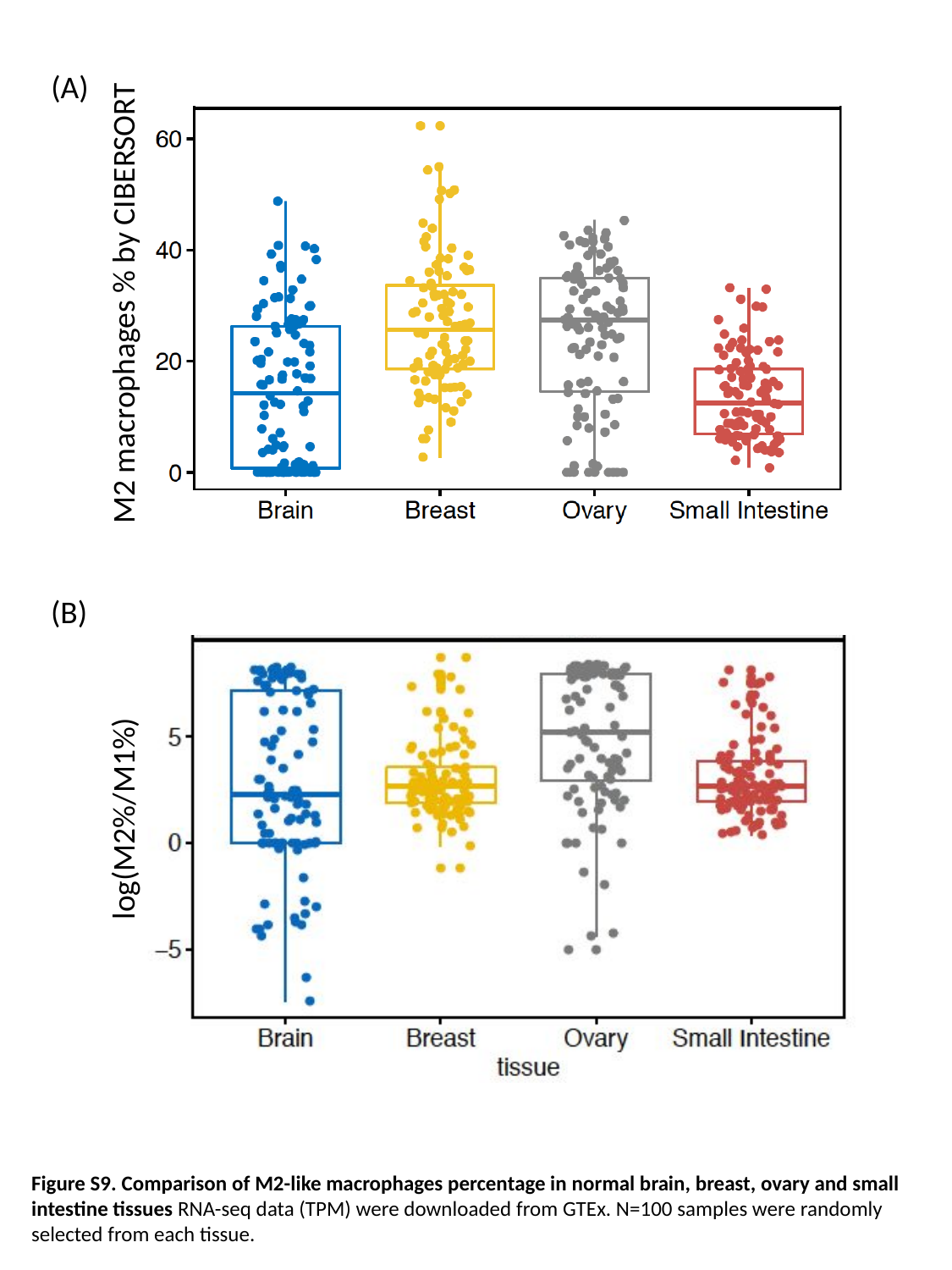

(A)
M2 macrophages % by CIBERSORT
(B)
log(M2%/M1%)
Figure S9. Comparison of M2-like macrophages percentage in normal brain, breast, ovary and small intestine tissues RNA-seq data (TPM) were downloaded from GTEx. N=100 samples were randomly selected from each tissue.

## Slide 15
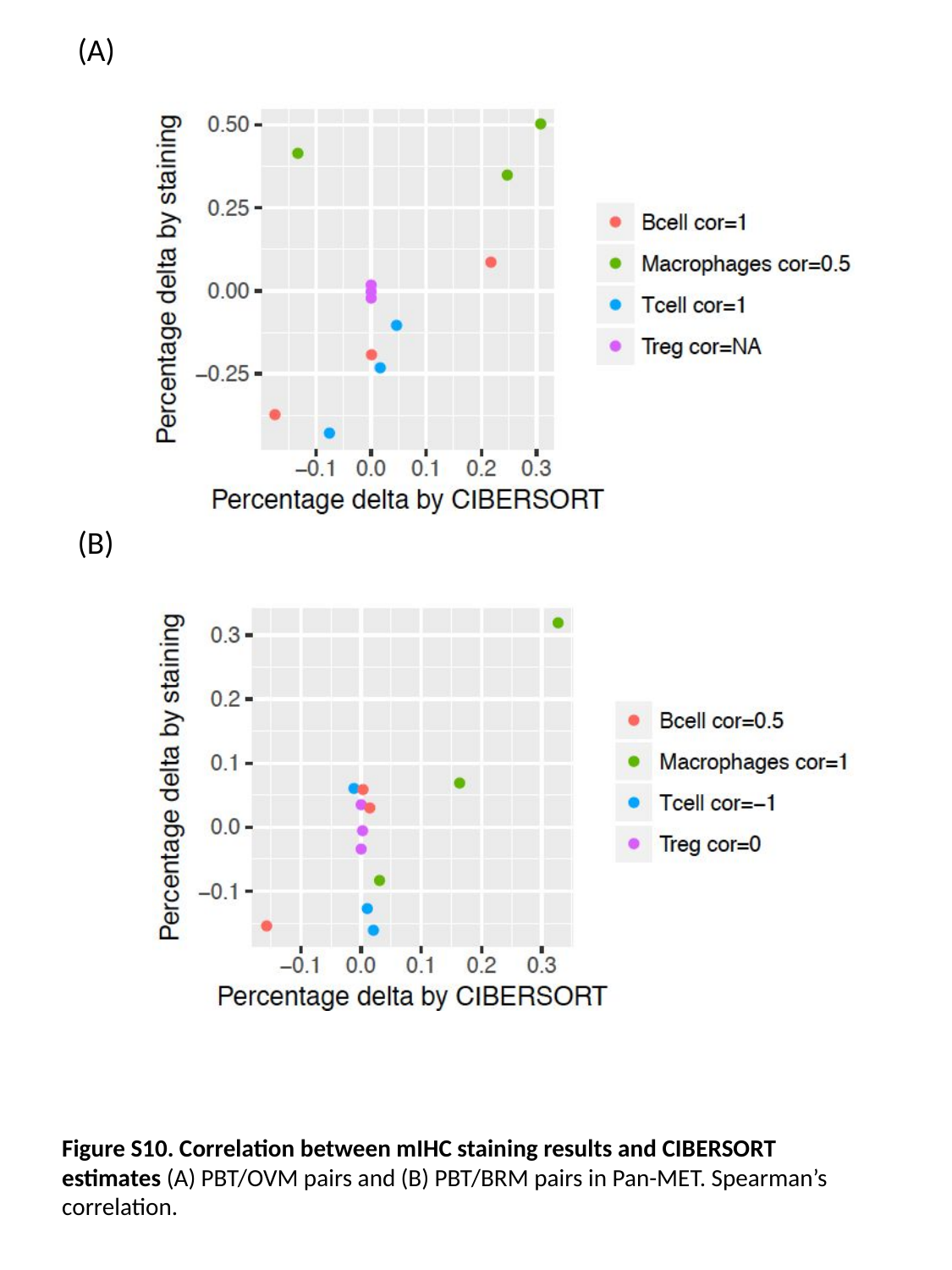

(A)
(B)
Figure S10. Correlation between mIHC staining results and CIBERSORT estimates (A) PBT/OVM pairs and (B) PBT/BRM pairs in Pan-MET. Spearman’s correlation.

## Slide 16
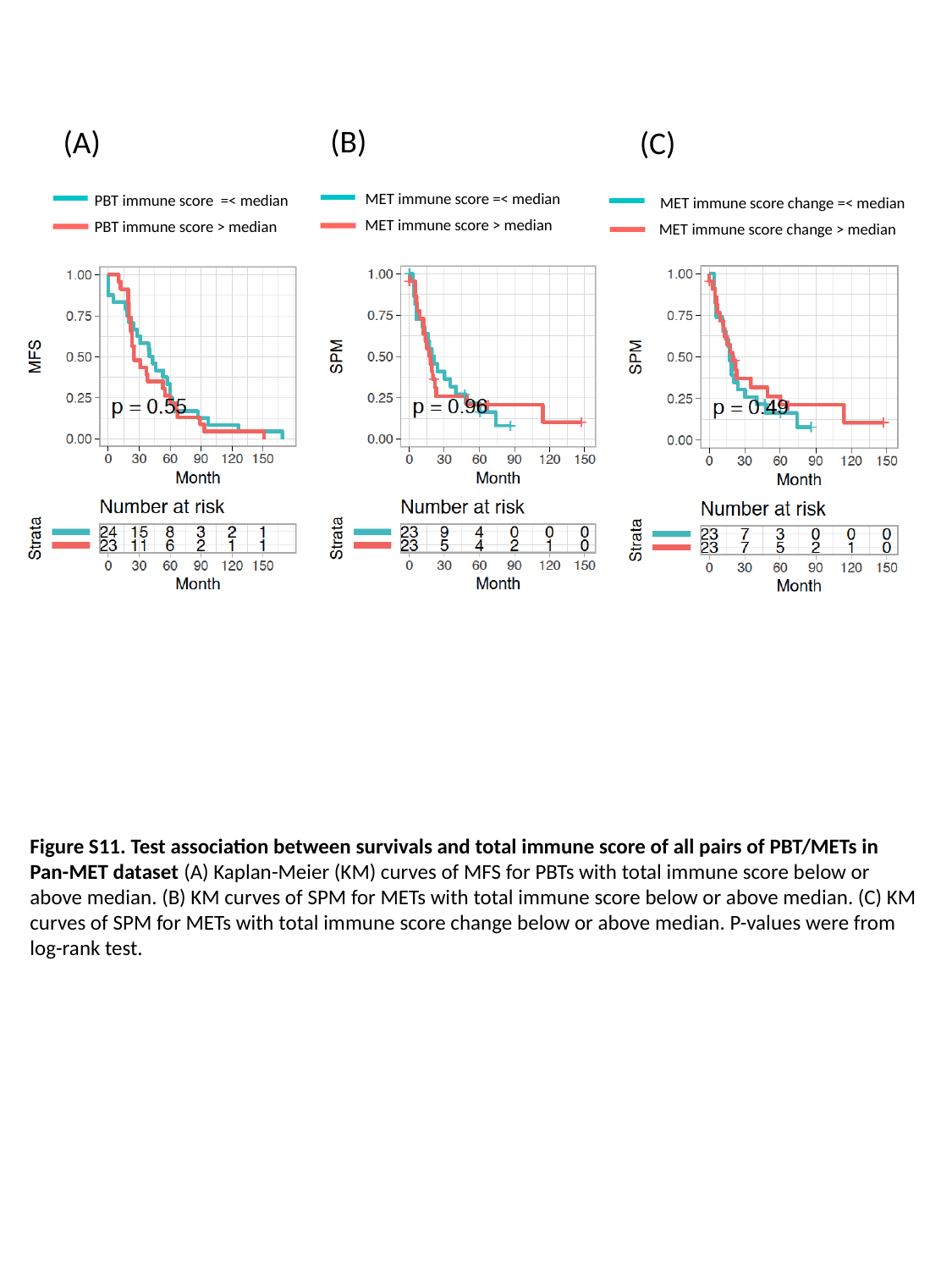

(B)
(A)
(C)
MET immune score =< median
PBT immune score =< median
MET immune score change =< median
MET immune score > median
PBT immune score > median
MET immune score change > median
Figure S11. Test association between survivals and total immune score of all pairs of PBT/METs in Pan-MET dataset (A) Kaplan-Meier (KM) curves of MFS for PBTs with total immune score below or above median. (B) KM curves of SPM for METs with total immune score below or above median. (C) KM curves of SPM for METs with total immune score change below or above median. P-values were from log-rank test.

## Slide 17
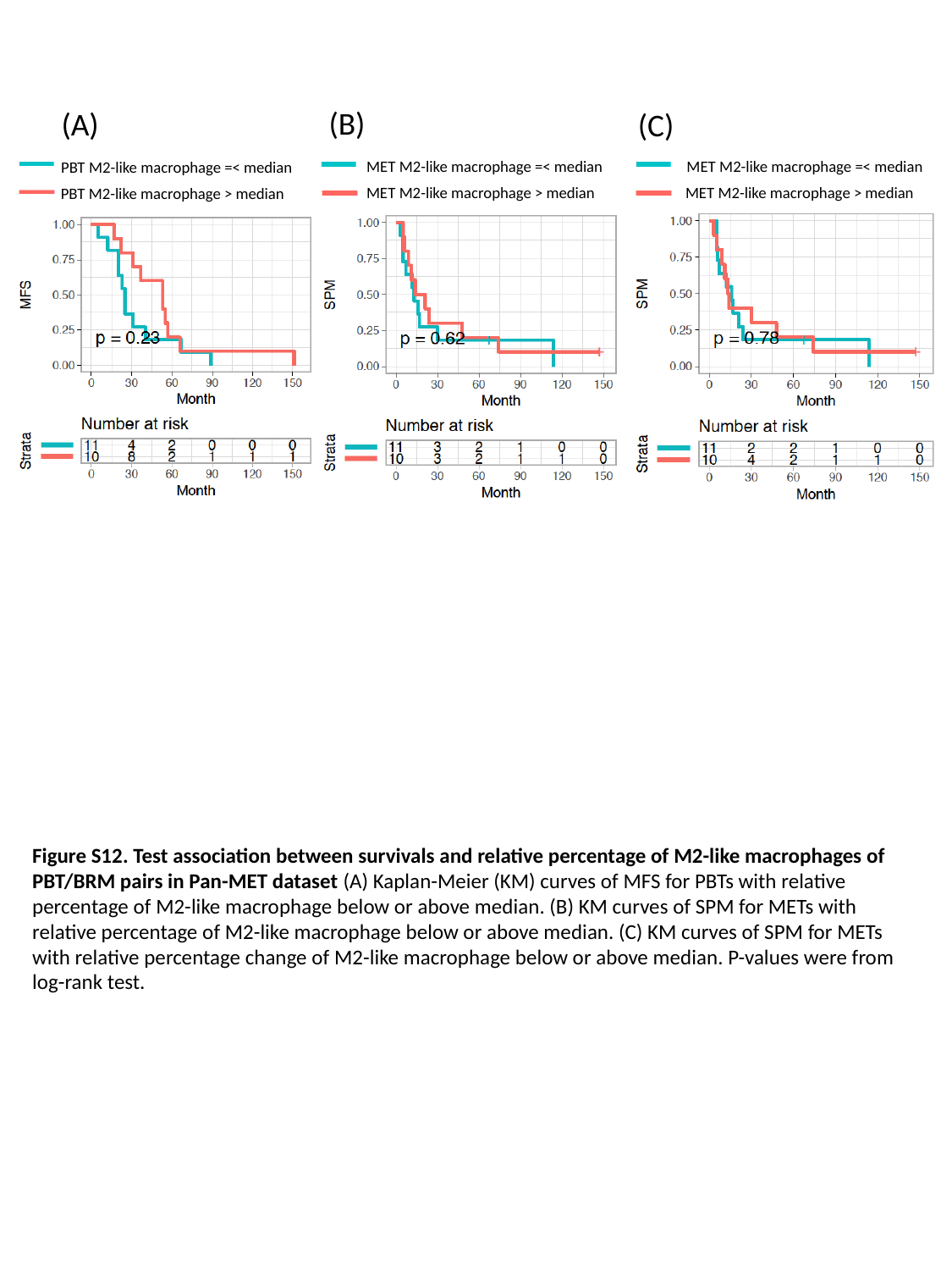

(B)
(A)
(C)
MET M2-like macrophage =< median
MET M2-like macrophage =< median
PBT M2-like macrophage =< median
MET M2-like macrophage > median
MET M2-like macrophage > median
PBT M2-like macrophage > median
Figure S12. Test association between survivals and relative percentage of M2-like macrophages of PBT/BRM pairs in Pan-MET dataset (A) Kaplan-Meier (KM) curves of MFS for PBTs with relative percentage of M2-like macrophage below or above median. (B) KM curves of SPM for METs with relative percentage of M2-like macrophage below or above median. (C) KM curves of SPM for METs with relative percentage change of M2-like macrophage below or above median. P-values were from log-rank test.
